# Immune escape pathways from the HBV core_18-27_ CD8 T cell response are driven by individual HLA class I alleles

**DOI:** 10.3389/fimmu.2022.1045498

**Published:** 2022-11-10

**Authors:** Andreas Walker, Tatjana Schwarz, Janine Brinkmann-Paulukat, Karin Wisskirchen, Christopher Menne, Elahe Salimi Alizei, Helenie Kefalakes, Martin Theissen, Daniel Hoffmann, Julian Schulze zur Wiesch, Mala K. Maini, Markus Cornberg, Anke RM Kraft, Verena Keitel, Hans H. Bock, Peter A. Horn, Robert Thimme, Heiner Wedemeyer, Falko M. Heinemann, Tom Luedde, Christoph Neumann-Haefelin, Ulrike Protzer, Jörg Timm

**Affiliations:** ^1^ Institute of Virology, University Hospital Düsseldorf, Heinrich Heine University Düsseldorf, Düsseldorf, Germany; ^2^ Institute of Virology, School of Medicine, Technical University of Munich, Helmholtz Zentrum München, Munich, Germany; ^3^ German Center for Infection Research (DZIF), Site Munich, Munich, Germany; ^4^ Department of Medicine II, University Hospital Freiburg, Faculty of Medicine, University of Freiburg, Freiburg, Germany; ^5^ Institute of Virology, University of Duisburg-Essen, University Hospital Essen, Essen, Germany; ^6^ Research Group Bioinformatics, Faculty of Biology, University of Duisburg-Essen, Essen, Germany; ^7^ Department of Medicine, University Medical Center Hamburg-Eppendorf, Hamburg, Germany; ^8^ German Center for Infection Research (DZIF), Site Hamburg, Hamburg, Germany; ^9^ Division of Infection and Immunity, Institute of Immunity and Transplantation, University College London, London, United Kingdom; ^10^ Department of Gastroenterology, Hepatology and Endocrinology, Hannover Medical School, Hannover, Germany; ^11^ German Center for Infection Research (DZIF), Site Hannover, Hannover, Germany; ^12^ Department of Gastroenterology, Hepatology and Infectious Diseases, University Hospital Düsseldorf, Heinrich Heine University Düsseldorf, Düsseldorf, Germany; ^13^ Institute for Transfusion Medicine, University Hospital Essen, University of Duisburg-Essen, Essen, Germany

**Keywords:** hepatitis B virus, CD8 T cell response, TCR-engineered T cells, immune escape, chronic infection

## Abstract

**Background and aims:**

There is growing interest in T cell-based immune therapies for a functional cure of chronic HBV infection including check-point inhibition, T cell-targeted vaccines or TCR-grafted effector cells. All these approaches depend on recognition of HLA class I-presented viral peptides. The HBV core region 18-27 is an immunodominant target of CD8+ T cells and represents the prime target for T cell-based therapies. Here, a high-resolution analysis of the core_18-27_ specific CD8+ T cell and the selected escape pathways was performed.

**Methods:**

HLA class I typing and viral sequence analyses were performed for 464 patients with chronic HBV infection. HBV-specific CD8+ T-cell responses against the prototype and epitope variants were characterized by flow cytometry.

**Results:**

Consistent with promiscuous presentation of the core_18-27_ epitope, antigen-specific T cells were detected in patients carrying HLA-A*02:01, HLA-B*35:01, HLA-B*35:03 or HLA-B*51:01. Sequence analysis confirmed reproducible selection pressure on the core_18-27_ epitope in the context of these alleles. Interestingly, the selected immune escape pathways depend on the presenting HLA-class I-molecule. Although cross-reactive T cells were observed, some epitope variants achieved functional escape by impaired TCR-interaction or disturbed antigen processing. Of note, selection of epitope variants was exclusively observed in HBeAg negative HBV infection and here, detection of variants associated with significantly greater magnitude of the CD8 T cell response compared to absence of variants.

**Conclusion:**

The core_18-27_ epitope is highly variable and under heavy selection pressure in the context of different HLA class I-molecules. Some epitope variants showed evidence for impaired antigen processing and reduced presentation. Viruses carrying such escape substitutions will be less susceptible to CD8+ T cell responses and should be considered for T cell-based therapy strategies.

## Introduction

Although, a prophylactic vaccine against hepatitis B virus (HBV) is available, persistent infections associated with chronic liver disease are still a global health problem. Chronic HBV-infections can be treated with IFNα or nucleot(s)ide analogues. However, a functional cure with persistently undetectable HBV-DNA and absence of liver inflammation is only rarely achieved (1, 2). Accordingly, there is growing interest in novel strategies leading to clearance of chronic HBV-infection. There is strong evidence that the immune response by CD8^+^ T cells contributes to sustained immune control of HBV infection (3–5). In turn, in chronic infection HBV-specific CD8^+^ T cells exhibit an exhausted phenotype with upregulation of inhibitory receptors and progressive dysfunction (4, 6). One of the proposed strategies for novel treatment interventions is therefore a combination of antiviral treatment with immune therapies including immune checkpoint inhibitors, T-cell inducing vaccines or transfer of TCR-grafted effector cells (4, 6, 7). These T cell-based therapies ultimately rely on presentation of viral epitopes by HLA class I molecules on infected hepatocytes. Given the sequence diversity of HBV, there is concern that viral variants in important epitopes may impact the efficacy of such novel treatment strategies.

We and others have shown previously, that viral variants in targeted CD8^+^ T cell epitopes impair the immune response (8, 9). Indeed, enrichment of certain viral sequence polymorphisms in epitopes presented by particular HLA class I molecules strongly suggest that epitope variants are selected as an immune evasion mechanism to escape from CD8^+^ T cell selection pressure. The region core_18-27_ represents an immunodominant HLA-A*02-restricted epitope that is well described in the literature (10–18). Interestingly, this epitope is a promiscuous binder and is also presented by HLA-B*35 and HLA-B*51 (9, 12). Given the high reproducibility of T cell responses against this epitope, it is considered an attractive target for T cell-based immune therapies. Here, we provide a high-resolution analysis of the HLA class I subtypes restricting the CD8^+^ T cell immune response to this epitope region, the selected HLA class I subtype-dependent pathways to immune escape and the responsible immune escape mechanisms.

## Material and methods

### Patients

In a multi-center effort for analysis of HLA class I-associated selection pressure 464 HBV patients were recruited at the Hepatology Units in Düsseldorf, Essen, Hamburg, Hannover, Freiburg (all Germany) and London (UK); (**Supplemental Table 1**). Only HBV genotype A and D infected patients were included. Informed consent was obtained from each patient, and the study protocol was approved by the local ethics committee of the Medical Faculty of Düsseldorf in accordance with the guidelines of the Declaration of Helsinki. Peripheral blood mononuclear cells (PBMCs) were isolated by Ficoll density gradient centrifugation (Biocoll; Biochrom) (9). DNA for HLA-typing was extracted from PBMCs using spin columns (Qiagen). High resolution (second field) HLA-A and HLA-B typing was performed by use of sequence-specific oligonucleotides (LABType™) methodology (19), provided by One Lambda (Thermo Fisher Inc.) at department of transfusion medicine of the University Hospital Essen.

### Amplification and sequence analysis of the HBV core region

Two-step nested PCRs were performed with GoTaq HotStart-Polymerase (Promega) Polymerase according to the manufacturer’s protocol and the following primer combinations for PCR-I: TS-1585_F_int (TTCGCTTCACCTCTGCACGT); TS-2419_R (GCGACGCGGNGATTGAGAYCT) and PCR-II: HBVCoreF (TGTCAACGACCGACCTTGAGG); TS-2397_R_int (CGTCTGCGAGGYGAGGGAGTTC). Per reaction 95 μl PCRI mixture containing 1x GoTaq polymerase buffer, 200 μM dNTPs (Bio-Budget), 0.5 μM each Primer and 1.25 units polymerase were mixed with 5 µl HBV-DNA. PCRII mixes were identical to PCRI except the final volume of 97 μl. PCR condition were 180 s at 94°C followed by 35 cycles each 30 s 95°C, 30 s 55°C and 120 s 72°C followed by 10 min at 72°C and hold at 10°C. Subsequently, three microliter of the first round PCR-product was used for the second round of nested-PCR with the same PCR conditions. PCR products were purified with the QIAquick PCR-Purification Kit (Qiagen, Hilden) and Sanger sequenced with sequencing primer HBVCoreF and TS-2397_R_int. For high-throughput sequencing Core-PCRs were sent to an external provider (SeqIt Kaiserlautern, Germany) and were sequenced on a MiSeq2 (Illumina) with a mean coverage of 15-20.000x. All obtained sequences were aligned with the software Geneious 10.2.6 (RRID : SCR_010519). Sequences were submitted to Genbank and are available under accession numbers (MZ043025-MZ043097; MZ097624-MZ097884).

### Analysis of HBV-specific CD8^+^ T cells

HBV-specific CD8^+^T cells were detected after antigen-specific expansion as previously described (20). Briefly, PBMCs were resuspended in RPMI medium containing 10% fetal calf serum and stimulated with individual peptides (1 µg/mL), anti-CD28/CD49d (0.5 µg/mL; BD Biosciences) and recombinant interleukin-2 (20 U/mL; Hoffmann-La Roche). On day 10 the cells were restimulated with prototype or variant peptides and secretion of interferon-γ (IFNγ) was analyzed by intracellular cytokine staining (ICS) and subsequent analysis on a FACS Canto (Becton Dickinson).

### Analysis of the HLA-restriction of antigen-specific CD8^+^ T cells

Partially HLA-matched PBMCs from healthy donors expressing either HLA-A*02:01, HLA-B*35:01 or HLA-B*51:01 were used as targets for antigen-specific CD8 T cells as effector cells, that have been expanded from PBMCs of HBV infected patients in the presence of the core 18-27 prototype peptide. The target cells were pulsed with the peptide (10µg/ml) overnight at 37°C and carefully washed five 5 times in PBS the next day. After washing 200.000 target cells were co-cultured with 200.000 effector cells in a 24 well plate for 5h followed by a standard ICS. Exogenously added peptide in the absence of target cells served as a positive control, target cells cultured overnight in the absence of peptide served as a negative control. The frequency of IFNγ+ CD8+ T cells in the negative control was considered as background and was subtracted from the results in the presence of peptide.

### Generation of HepG2 cells stably expressing the HBV-core-mCherry fusion protein

For generation of stable cell lines the GFP sequence in the bicistronic pWPI vector (kindly provided by D. Trono, EPFL) was replaced by a Blasticidin-S deaminase and the synthetic sequence for an HBV core_aa1-183_-mCherry fusion protein (Eurofins genomics) was inserted by Gibson assembly (NEB). The different variants in the core_18-27_ sequence were introduced by site-directed mutagenesis. Generation of lentiviral pseudoparticles was described previously (21). In brief, 2.5×10^6^ 293T cells were co-transfected with pWPI-core-mCherry-BSD, pCMVR8.74 and pMD2.G using Mirus TransIT^®^-LT1 (Mirus) transfection reagent and pseudoparticles were harvested after 48h and 72h. For transduction 2x 10^6^ HepG2 cells were spinoculated (MOI 1-5) for 30min at 37°C and 700g and seeded in DMEM. Twenty-four hours after infection cells were selected with 25 µg/ml Blasticidin (*In vivo*gen). HEK293T and HepG2 cells were cultured in Dulbecco’s modified Eagle Medium (2mML-glutamine, 10 mM HEPES, nonessential amino acids, 100 U of penicillin/ml, 100 µg of streptomycin/ml, 10% fetal calf serum).

### Analysis of the CD8^+^ T cell response against endogenously processed antigens

Stable HBV-core-mCherry expressing HepG2 cells were seeded at a density of 2x10^5^ cells/well in a 6-well Plate in DMEM without blasticidin. The next day, 2x10^5^HBV core-specific CD8^+^ T cells were co-cultured for 4h with the HepG2 cells before IFN-γ was analyzed by ICS. As control HepG2 cells were incubated with 10 mg/ml prototype or variant peptide overnight, washed extensively and were then used as targets as above described.

## Results

### Influence of the HLA class I subtype on the CD8^+^ T cell response against the core_18-27_ region

**Figure 1:**
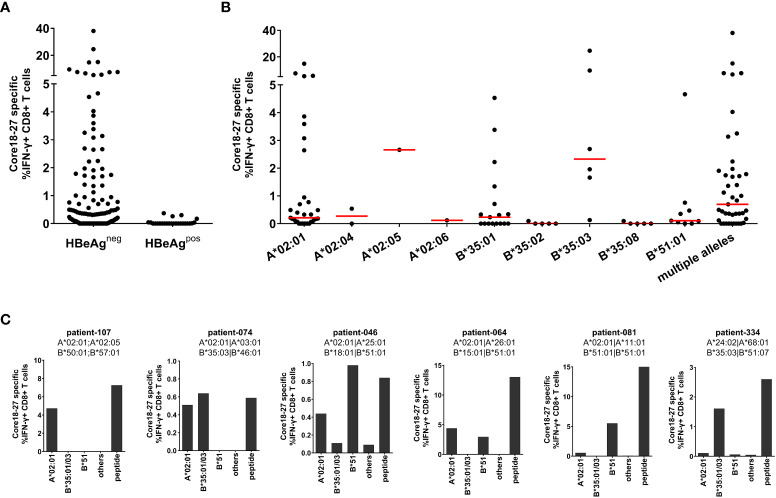
The core_18-27_ epitope is restricted by multiple HLA class I molecules. PBMCs from 154 patients with chronic HBV infection were stimulated for 10 days in the presence of the core_18-27_ peptide and re-stimulated for 5h before IFNγ production was determined by ICS. Depicted are IFNγ producing HBV core 18-27 specific CD8 T cells, the IFNγ response from non-stimulated cell was subtracted **(A)** IFNγ responses according to the HBeAg status. **(B)** IFNγ responses according to the HLA-type of the HBeAg negative patients from **(A)**. Patients depicted with single HLA-types had no other relevant HLA-type (e.g. A*02:01 are patients negative for HLA-B*35:01/03 or HLA-B*51 and vice versa), patients with “multiple alleles” had at least two relevant HLA-alleles (e.g. A*02:01 and B*51). **(C)** The HLA-restriction of antigen-specific CD8^+^ T cells was determined with partially HLA-matched peptide-pulsed target cells expressing only HLA-A*02:01 (not B*35 or B*51), B35:01/03 (not A*02:01 or B*51), HLA-B*51 (not A*02:01 or B35), others (not A*02:01, B*35 or B*51). As control core18-27 peptide was directly added to the culture (peptide). The frequency of IFNγ -producing CD8^+^ T cells was determined by ICS and a representative example is shown in **Supplemental Figure S3**.

The HLA-A*02 restricted epitope in HBV core_18-27_ is frequently targeted by patients and is a prime target for T cell therapy (22). Since there is evidence, that the core_18-27_ region is also immunogenic in the context of HLA-A*02, HLA-B*35 and HLA-B*51 alleles (11, 12, 20), the HLA-restriction was analyzed in more detail. Therefore, the CD8^+^ T cell immune response against HBV core_18-27_ was determined in 154 chronic HBV infected patients. PBMCs were cultured for 10 days in the presence of the core_18-27_ peptide and the frequency of IFNγ-producing cells upon re-stimulation with the peptide was determined by flow cytometry. Notably, in this analysis only HBeAg negative patients showed detectable CD8^+^ T cell responses directed against this epitope (**Figures 1A**; **Supplemental Figure S2**). Accordingly, in a subsequent analysis of the relevant HLA class I-alleles for the CD8^+^ T cell response, only HBeAg negative patients were included. Consistent with promiscuous binding and presentation of the peptide, antigen-specific production of IFNγ by CD8^+^ T cells was detected in patients carrying HLA-A*02, HLA-B*35 and HLA-B*51 (**Figure 1B**).

Importantly, there were differences in the CD8^+^ T cell response between different HLA class I-subtypes. In the context of HLA-B*35 only patients carrying the subtype HLA-B*35:01 and B*35:03 mounted an immune response against the epitope whereas patients with HLA-B*35:02 and HLA-B*35:08 did not. The most robust and reproducible CD8^+^ T cell response was detected in HLA-B*35:03 positive patients. In the group of HLA-A*02 positive patients the most frequent subtype was HLA-A*02:01. Although the number of patients with non-HLA-A*02:01 subtypes was too low to draw solid conclusions, at least one patient with HLA-A*02:05 also mounted a robust immune response against the epitope. In the group of HLA-B*51 positive patients, all analysed patients had the subtype HLA-B*51:01. Interestingly, the group of patients carrying multiple relevant HLA class I-alleles showed a higher median of the CD8^+^ T cell response (0.69%) than HLA-A*02:01 (median 0.21%), HLA-B*35:01 (median 0.23%) and HLA-B*51:01 (median 0.10%) positive patients (**Figure 1B**), suggesting that presence of multiple relevant HLA class I-alleles might be associated with a more robust immune response.

The restricting HLA-molecule was further analysed in six patients carrying one or two relevant HLA class I alleles, with heterologous partially HLA-matched peptide-pulsed target cells. These analyses revealed, that in some patients two distinct CD8^+^ T cell immune responses were detectable, each restricted by different HLA class I molecules (**Figure 1C**, patients 074, 046 and 064). In other cases, only one HLA class I molecule dominated the response despite presence of a second relevant allele (**Figure 1C**, patient 081). Taken together, the core_18-27_ region is presented by multiple HLA class I subtypes with the strongest evidence for CD8^+^ T cell immune responses in the context of HLA-A*02:01, HLA-B*35:01, HLA-B*35:03 and HLA-B*51:01.

### Sequence analysis of the core_18-27_ region

Earlier studies suggested that naturally occurring sequence variants in core_18-27_ were functionally associated with immune escape (8) and were a product of selection pressure in HLA-A*02-positive individuals (9). To decipher the individual effect of each HLA-molecule on selection pressure, we analyzed core sequences from 409 HBV-patients, infected with genotype A or D. The cohort included 83 patients positive for HLA-A*02:01 (not other HLA-A*02-subtypes, B*35 or B*51), 13 patients positive for HLA-A*02:xx (not HLA-A*02:01, B*35 or B*51), 62 patients positive for B*35:01 or B*35:03 (not A*02 or B*51), 35 patients positive for HLA-B*51:01 (not A*02 or B*35), 104 patients with combinations of these four HLA class I alleles and 112 patients negative for all four HLA alleles.

**Figure 2:**
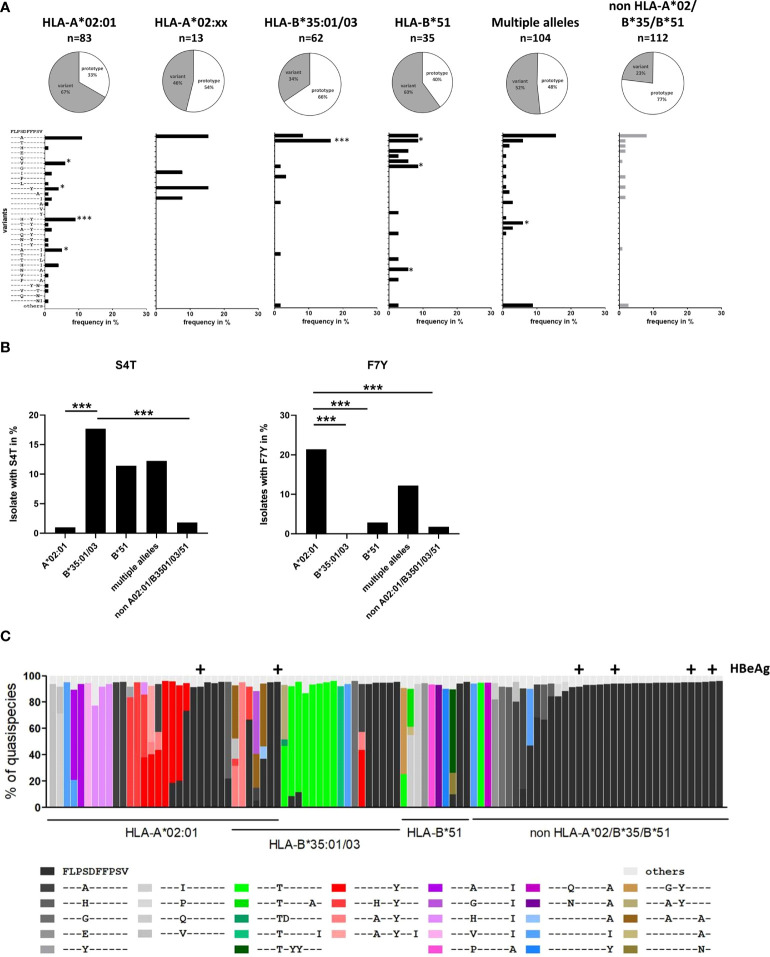
Frequency of sequence polymorphisms in the CD8^+^ T-cell epitope HBV core_18-27_. **(A)** 717 bp amplicon from the core region was amplified and Sanger sequenced from 409 patients with known HLA-type and chronic HBV infection (genotype A or D). The pie charts show the frequencies of core_18-27_ variants in patients with the depicted HLA-type. The lower panels show the frequencies of individual epitope variants for isolates from patients carrying only HLA-A*02:01, HLA-A*02 subtypes others than A*02:01 (A*02:xx), only HLA-B*35:01/03, only HLA-B*51:01, multiple alleles or none of the relevant alleles. Epitope variants that are significantly more frequent compared to patients lacking any of the relevant HLA class alleles by Fisher’s exact test are indicated (*p < 0.05; ***p < 0.001). **(B)** Frequency of sequence polymorphisms in the core_18-27_ epitope. Cumulative frequencies of sequence polymorphism were calculate regardless of other sequence polymorphism in the core 18-27 epitope for S4T (left) and F7Y (Right). **(C)** Analysis of HLA-associated frequencies of individual variants in the core_18-27_ region within the quasispecies. Amplicons of 96-randomly chosen patients from **(A)** were submitted to high-throughput sequencing with a mean coverage of 15-20.000x. Reads were aligned to the patient-specific reference sequence generated by Sanger sequencing. Each bar represents the core_18-27_ region of one patient with the individual epitope variants being color coded as indicated. Only variants with frequencies >5% of the quasispecies are shown. Samples from HBeAg-positive patients are marked above the column with (+).

Overall, the epitope region is highly polymorphic with frequent substitutions predominantly in positions 4, 7 and 10 of the epitope (**Figure 2A**). The frequency of any variation from the prototype sequence in the absence of the relevant HLA class I alleles was 23% (26 of 112) with the S4A substitution being the most frequent (8%). Notably, the frequency of the S4A substitution was not significantly different in the presence of HLA*02, HLA-B*35:01/03 or HLA-B*51:01 suggesting that this substitution was not driven by CD8 T cell pressure in the context of these alleles. However, the frequency of sequences with other substitutions in the epitope region was significantly enriched in patients with these relevant HLA class I alleles. For example, in HLA-B*35:01/03 positive patients an S4T substitution was reproducibly selected. Here, ten of 62 HLA-B*35:01/03 positive patients (16.1%) had the S4T substitution. This was in contrast to HLA-A*02 positive patients and patients lacking any of the relevant alleles, where this substitution was not or only rarely observed (p<0.0001) (**Figures 2A, B**). Of note, also in HLA-B*51:01 positive patients substitutions were preferentially selected in position 4 of the epitope, but in addition to S4T, other mutations such as S4G, S4V and other rare substitutions were observed that were nearly undetectable in the absence of any of the relevant alleles. In contrast, in HLA-A*02:01 positive patients the F7Y substitution alone or in combination with substitutions in position 4 (e.g. S4H) was significantly enriched compared to HLA-B*51 or HLA-B*35:01/03 positive patients or patients lacking the relevant HLA alleles (p<0.05 and p<0.0001; **Figures 2A, B**). The selective enrichment of the S4T substitution in HLA-B*35:01/03 positive patients and of the F7Y substitution in HLA-A*02:01 positive patients suggests that the pathways to CD8^+^ T cell escape differ between the presenting HLA class I molecules. Phylogenetic analysis showed no founder effect or genotype association of the substitutions (**Supplemental Figure S1**) supporting the conclusion that the variants were selected by individual immune pressure.

To analyze the epitope diversity at the quasispecies level, the core-region was amplified from 96 randomly-chosen patients and sequenced on a MiSeq Illumina platform with a median coverage of 12,358-fold (range 1,028 - 52,336-fold). The frequency of individual epitope variants within the quasispecies is shown in **Figure 2C**. The prototype sequence is shown in black and each variant is color-coded as indicated. HBeAg positive patients are marked above the columns. For clarity, variants including the S4T substitution are coded in shades of green. Variants including the F7Y substitution are coded in shades of red. Variants with two substitutions in position 4 and 10 are coded in shades of pink. Interestingly, detection of multiple epitope variants within the quasispecies was common in patients carrying any of the relevant HLA class I alleles, which would be consistent with continuous selection. In line with the population sequence data, variants were enriched in patients carrying any of the relevant HLA class I alleles and there was a clear difference between the HLA-types. In HLA-A*02:01 positive patients, variants with the F7Y substitution (shades of red) were enriched, while the variant S4T did not occur at all, not even in minor frequencies. In HLA-B*35:01/03 positive patients variants with the S4T substitution (shades of green) were more frequent and often the major variant.

**Figure 3:**
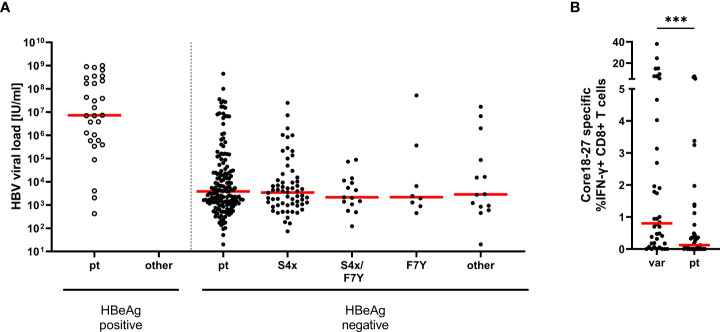
Association between HBV-DNA and variants in the epitope core_18-27_. **(A)** The HBV-DNA concentration is shown for 280 treatment-naive patients (genotype A and D) according to the absence and presence of substitutions in position 4 and 7 of the epitope. Patients were grouped according their epitope sequence and their serological HBeAg status as indicated. pt = prototype, S4X = any variant at position 4. **(B)** core_18-27_-specific CD8^+^ T cells responses from patient samples shown in **Figure 1A**, in HBeAg negative patients according to their epitope sequence (**Supplemental Table 1**); var = any variation in the core 18-27 epitope; pt = prototype. Statistical significance was calculated with the Mann-Whitney-test (p = 0.0001). *** = p<0.001.

Absence or lower frequencies of substitutions in patients lacking the relevant HLA-molecules suggests negative selection of epitope variants in the core18-27 region in the absence of immune pressure. We therefore tested, if the substitutions in the epitope region were associated with lower HBV-DNA concentrations in patients. Therefore, HBV-DNA concentrations and the core18-27 sequence were analyzed in treatment-naïve patients (n=280). The patients were classified according to their serological HBeAg status. As expected, the viral load was significantly higher in HBeAg-positive patients than in HBeAg-negative patients (**Figure 3A**). Interestingly, in the HBeAg positive group all isolates carried the prototype sequence consistent with absence of selection pressure by CD8 T cells. In HBeAg negative patients, there was no significant difference in viral load between prototype and the different variants. It was also addressed, if epitope variants associated with a distinct magnitude of the CD8 T cell response after 10 days of antigen-specific expansion. As no CD8 T cell responses and no epitope variants were detected in HBeAg positive patients (**Figure 1A**), HBeAg positive individuals were excluded from the analysis to avoid a bias based on the HBeAg status (**Figure 3B**). Notably, despite exclusion of HBeAg positive patients, infection with a variant was associated with a significantly stronger T-cell response compared to infection with the prototype (**Figure 3B**; p=0.0006), consistent with selection pressure in these patients.

### Cross-reactivity of CD8^+^ T cells with variants of the core_18-27_ epitope

**Figure 4:**
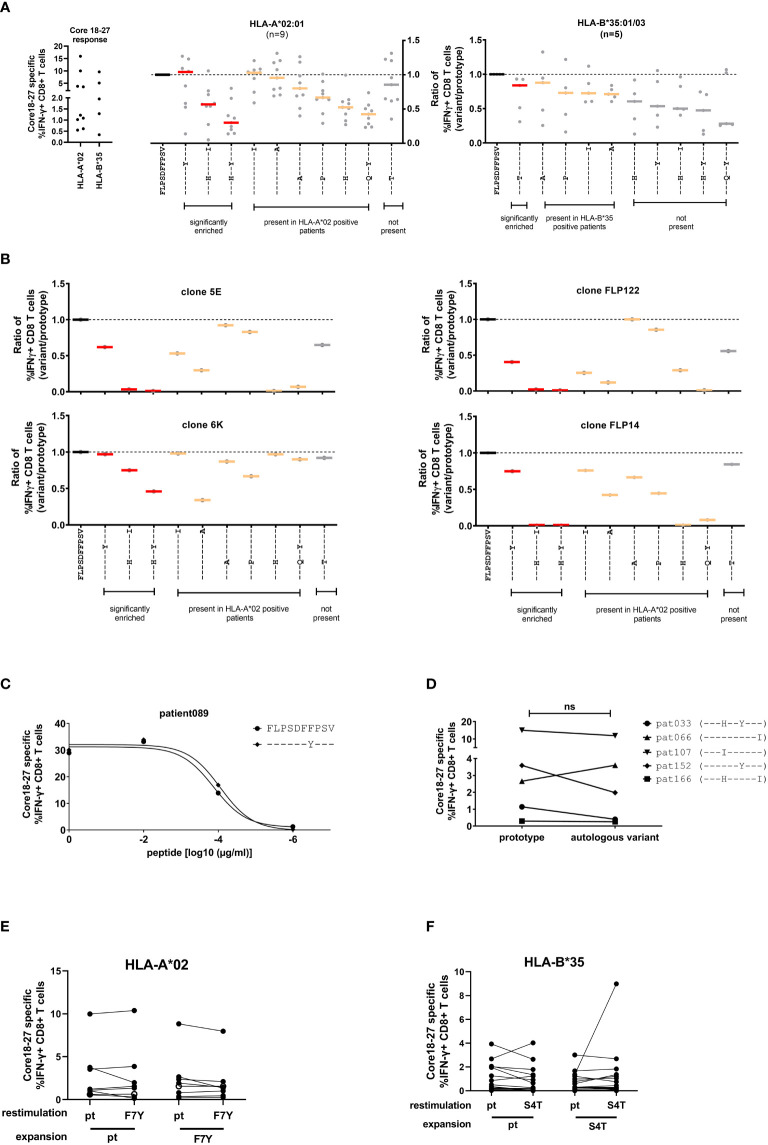
Cross-reactivity of core_18-27_-specific CD8^CD8+^ T cells with naturally occurring sequence variants. After antigen-specific expansion for 10 days, core_18-27_-specific CD8^+^ T cells were restimulated with the most common variants in naturally occurring isolates from **Figure 2** and IFNγ-producing CD8^+^ T cells were determined by ICS. For comparison the responses were normalized to the prototype (FLPSDFFPSV) response. **(A)** *Left:* the core 18-27 specific CD8 T cell response of the patients used for the cross-reactivity analysis before normalization. Middle/Right: Cross-reactivity of core_18-27_-specific CD8^+^ T cells with peptide variants in HLA-A*02:01 or HLA-B*35:01/03 positive patients. The response against variants with statistical support for selection on the sequence level (**Figure 2**) are marked in red. **(B)** Cross-reactivity of HLA-A*02 restricted grafted T effector cells. **(C)** The CD8^+^ T-cell response against different concentrations of the prototype peptide or the F7Y variant determined by ICS. **(D)** The core 18-27 CD8^+^-T cell response against the prototype or the autologous viral sequence present in the corresponding patient. **(E)** Expansion of core 18-27 CD8^+^-T cells with the prototype or F7Y-variant peptide and restimulation with the indicated peptide in HLA-A*02 positive patients (n=8) **(F)** Expansion of core 18-27 CD8^+^-T cells with the prototype or S4T-variant peptide and restimulation with the indicated peptide in HLA-B*35 positive patients (n=20). ns = not significant p >0.05.

To analyze the impact of epitope variation on the CD8^+^ T cell response, PBMCs were expanded in the presence of the prototype epitope and restimulated at day 10 with different epitope variants. The peptides included significantly enriched variants in the presence of HLA-A*02:01 or HLA-B*35:01/03 and additional variants that were detect in the presence of these alleles without statistical support for selection as well as variants that were not detected in the presence of these allele. The frequency of IFNγ-producing cells was determined by flow cytometry (**Figure 4A**, left panel and **Supplemental Figure S4**) and the results were normalized to the prototype response (**Figure 4A**, middle and right panel). In line with functional immune escape from HLA-A*02:01-restricted CD8^+^ T cells the double variant S4H/F7Y and to a lesser extent the S4H/V10I variant showed substantial reduction in the IFNγ response (**Figure 4A**). Interestingly, the variant carrying the single F7Y substitution, that was reproducibly selected in HLA-A*02:01 positive patients, showed no clear tendency towards functional immune escape in this assay. Notably, the same degree of cross-reactivity of the F7Y variant was observed, when serial dilutions of the peptide were tested, suggesting that the functional avidity was not impaired by the variant peptide (**Figure 4C**). Also in HLA-B*35:01/03 positive individuals, the selected S4T substitution was highly cross-reactive (**Figure 4A**). Importantly, there was substantial variation between individuals regarding the degree of cross-reactivity with epitope variants. Therefore, the cross-reactivity of individual T cell receptors was determined by utilizing CD8^+^ T cells transduced with defined TCRs from donors with an HLA-A*02:01-restricted T cell response against this epitope (16, 23) (**Figure 4B**; **Supplemental Figure S5**). Indeed, the cross-reactivity profiles differed between individual TCRs. The TCR 6K showed substantial cross-reactivity with the selected variants including the variants carrying double substitutions. In contrast, the other TCRs were more sensitive to substitutions in the epitope. Interestingly, even here, the F7Y variant was reactive for all four TCRs, suggesting that it does not represent an optimal TCR-escape mutation.

Given the individual differences in the cross-reactivity patterns between patients and TCRs, we hypothesized, that the selected viral variant in a given host is the result of a highly individualized process. We therefore tested in patients with chronic HBV-infection the cross-reactivity with the individual’s autologous virus (**Figure 4D**). Again, in most cases we did not find clear evidence for functional immune escape with the variants. We also tested the ability of the variant peptides F7Y and S4T to expand antigen-specific CD8 T cells in HLA-A*02:01 or HLA-B*35:01 positive individuals (**Figures 4E, F**; **Supplemental Figure S6**). Again, there was no clear reduction of the IFNγ response by the variant. The frequency of antigen-specific CD8 T cells expanded with the variant peptide was at the same level compared to prototype and showed the same degree of cross-reactivity (**Figures 4E, F**). Of note, in line with previous results (24, 25), there were also no differences regarding the maturation state and expression of inhibitory receptors on antigen-specific CD8^+^ T cells when compared between patients with or without escape mutations (data not shown).

Taken together, variants carrying two substitutions selected in the context of HLA-A*02:01 showed functional evidence for immune escape. Interestingly, despite strong statistical evidence for selection of the F7Y variant in HLA-A*02:01-positive patients and the S4T variant in HLA-B*35:01/03 positive patients, there was no functional evidence for immune escape from assays with exogenously added peptide. This suggests that neither HLA class I-binding nor TCR binding to the HLA class I/peptide complex was impaired by these two variants.

### Influence of core_18-27_ epitope variants on processing and presentation by HLA-A*02:01

**Figure 5:**
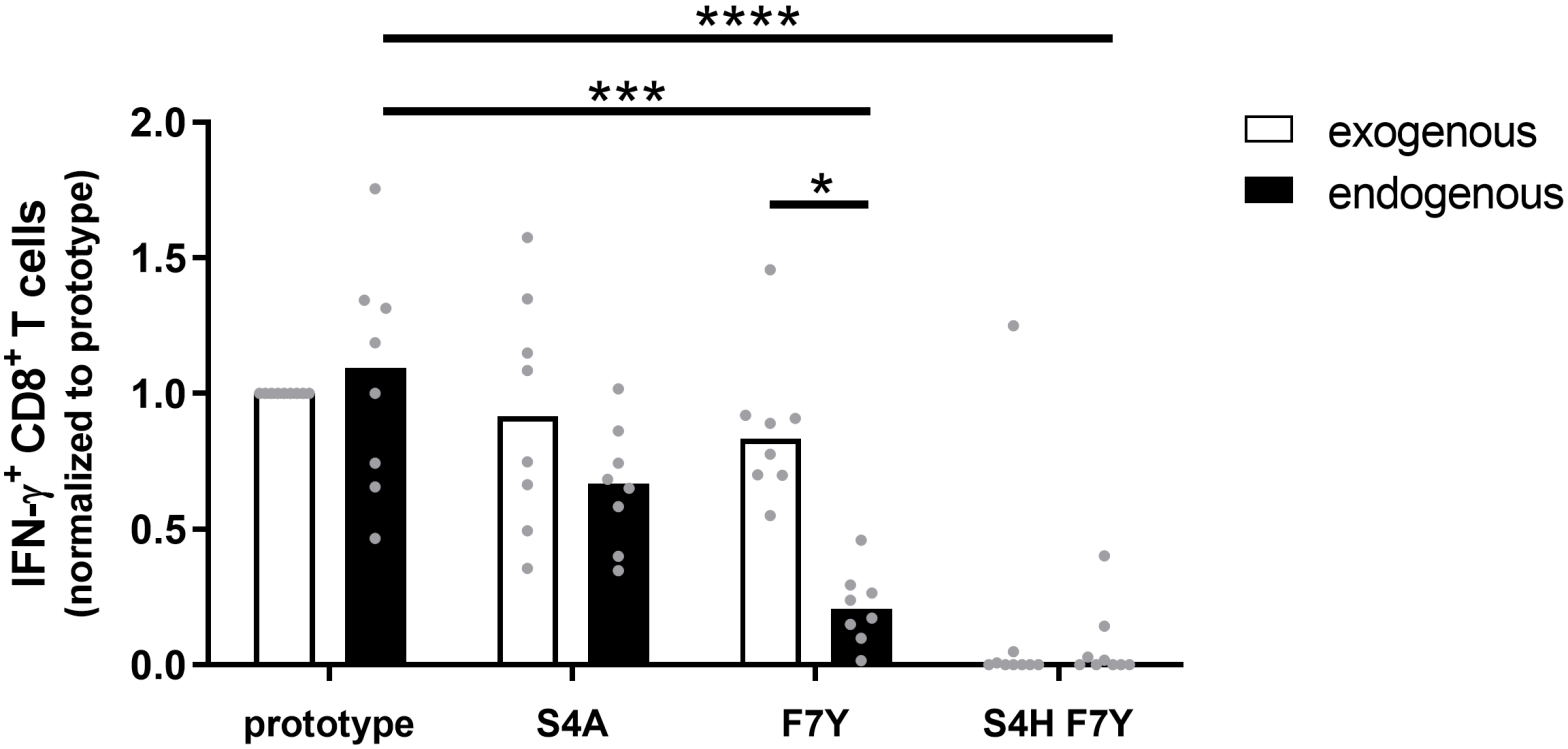
CD8^+^ T cell response against the endogenously processed epitope core_18-27_. Target cells stably expressing the HBV core protein were generated by transduction of HepG2 cells with prototype or variant HBV core (aa1-183) fused to mCherry. Effector T cells from different HLA-A*02:01-positive donors were expanded for 10 days in the presence of the core_18-27_ peptide FLPSDFFPSV. HBV core expressing HepG2 cells (endogenous) or peptide-pulsed HepG2 cells (exogenous) were used as targets for re-stimulation of core_18-27_-specific effector CD8^+^ T cells in an effector:target ratio of 1:1 followed by an ICS. The IFNγ response was normalized to the response against HepG2 cells with prototype peptide. P-values were calculated by One-way Anova with a Bonferroni’s Multiple Comparison Test. *p < 0.05; ***p < 0.001; ****p < 0.0001.

Given the statistical support for selection of the F7Y and S4T substitution in the presence of HLA-A*02:01 or HLA-B*35:01/03, the absence of a clear functional impact on CD8^+^ T cell cross-reactivity was unexpected. Our functional assays as well as the predicted binding affinities suggested that binding to the HLA class I molecule is not impaired when the synthetic peptide is exogenously added to the assay. This assay, however, does not include endogenous processing and presentation of the peptide from the viral protein and it has been previously shown that sequence variants within the epitope as well as in the epitope flanking region can associate with altered epitope processing (26–28). To address the influence of sequence variants on epitope processing, HepG2 cells were stably transduced with different variants of the core protein. HepG2 naturally express HLA-A*02:01 on their surface and the epitope is processed from endogenous protein repertoire. The complete core protein (genotype D) was fused to mCherry to compare expression levels and the frequency of mCherry positive cells and the mean fluorescence intensity (MFI) were analyzed before each experiment (**Supplemental Figure S7**). These cell lines were used as targets for core_18-27_-specific HLA-A*02:01-restricted CD8^+^ T cells and the IFNγ response was determined by flow cytometry. **Figure 5** shows the results of nine independent experiments. An example and the gating strategy is outlined in **Supplemental Figure S8**. The same frequency of IFNγ-positive cells was detected when target cells were transfected with the prototype core protein or when they were pulsed with the synthetic prototype peptide, indicating that the prototype epitope can be processed from the fusion protein. The frequent S4A substitution, showed a slightly but not significantly reduced IFNγ production when endogenously processed. In line with our previous results, the variant with the double S4H-F7Y substitution showed a substantially impaired immune response, when the synthetic peptide was exogenously added to the assay or endogenously processed. In contrast, the exogenously added F7Y variant peptide did not impair the immune response, however, here, the frequency of IFNγ-producing CD8^+^ T cells was reduced 4-fold when the variant epitope was endogenously processed, suggesting that the F7Y mutation interferes with correct antigen processing.

## Discussion

The core region 18-27 is one of the immunodominant targets of the CD8^+^ T cell response and is considered a prime target for future T cell based immune therapies. Here, we performed an in-depth analysis of the presenting HLA class I molecules, the sequence diversity of the epitope between and within individuals, the HLA-dependent pattern of selection pressure and propose a mechanisms of immune escape.

There are different reports suggesting promiscuous binding and presentation of the core_18-27_ epitope by different HLA class I molecules. In MHC-class-I binding assays, binding to a variety of HLA-class I-molecules including HLA-A*02, B*07, B*35, B*44, B*51 (13) has been described. Moreover, beyond CD8^+^ T-cell responses against core_18-27_ in the context of HLA-A*02, earlier studies demonstrated also CD8^+^ T cell responses restricted by HLA-B*35 (11, 13) and HLA-B*51 (11, 12). Notably, despite reported binding to HLA-B*07 (11, 13) we did not detect any robust responses in HLA-B*07-positive patients with chronic infection (data not shown). In the context of T-cell based immune therapies promiscuous binding and presentation of epitopes by different HLA class I molecules may have the advantage of broader HLA coverage in the target population. In our cohort, 62.2% of the patients carried at least one relevant HLA class I allele consistent with a high frequency of individuals mounting a response against this particular epitope.

Interestingly, despite promiscuous binding of the core_18-27_ epitope to different HLA class I types, we observed an impact of the HLA class I-subtype on the ability to mount a CD8^+^ T cell response. This was most evident in the context of HLA-B*35, as here CD8^+^ T cell responses were only detected when the epitope was presented by HLA-B*35:01 or HLA-B*35:03 but not when presented by HLA-B*35:02 or HLA-B*35:08. The exact subtype-specific restriction was less clear in the context of HLA-A*02. Most HLA-A*02-subtypes, other than HLA-A*02:01, were in the “multiple alleles” group and it is therefore difficult to draw solid conclusions for these less frequent alleles. Notably, it was previously described, that the core_18-27_ epitope is immunogenic in HLA-A*02:01 but not in HLA-A*02:03 (14), suggesting that also here, subtype-specific differences play a role for the immune response. Collectively, these results highlight that high-resolution HLA class I-typing is required for optimal results of T cell-based therapies.

In line with selection pressure by CD8^+^ T cells in the context of HLA-A*02:01, HLA-B*35:01/03 and HLA-B*51:01 we observed increased frequencies of substitutions in the epitope region in patients carrying any of the relevant HLA class I-alleles compared to patients lacking these alleles. In a previous study, we could show, that viral substitutions in the core_18-27_ epitope are significantly enriched in HLA-A*02 positive patients (9). Here, we extended the analysis and provide evidence, that the core_18-27_ region is also under selection pressure when presented by other HLA class I-molecules. Notably, variants were predominantly detected in HBeAg-negative patients and not in HBeAg-negative patients. This is in line with the concept of a tolerogenic function attributed to the HBeAg [reviewed in (29)]. In transgenic mouse model, mice carrying a core-specific TCR lacked an immune response against the HBcAg when the HBeAg was expressed at high levels (30). In turn, when the HBeAg was expressed from a promotor with low activity, the mice mounted a weak core-specific immune response and when no HBeAg was present, a strong and robust T cell response was observed. Although the mechanism of the putative immunomodulatory HBeAg function is not understood, a consequence may be lack of immune selection of CD8 T cell epitope variants in the presence of HBeAg as observed in our study. Notably, epitope variants are also absent during acute resolving HBV infection. In a previous study the core region from HLA-A*02-positive patients with acute HBV infection was sequenced (24). Here, eight patients infected with genotype A or D were longitudinally followed and the earliest and last viremic sample was sequenced. All patients carried the prototype sequence in the core_18-27_ epitope at the earliest time point. For five patients, a second time point between 2-4 weeks later was sequenced and in none of them a variant was selected before they continued to spontaneously resolve the infection.

The role of viral escape from the CD8^+^ T cell response has been debated in the context of hepatitis B. Early studies by Rehermann et al. concluded that mutational escape does not contribute to immune evasion of HBV, based on the observation that no epitope variants were observed in patients with HBV infection (17). However, the studied cohort was dominated by HBeAg positive infections, where we also do not see any evidence immune selection and the T cell response against epitope variants was not analyzed. Notably, the observed V10I substitution represents the prototype residue in HBV genotypes B/C, which are predominant in China. In line with a potential immune evasive effect, a prior study reported a reduced T cell response against the core_18-27_ epitope in a Chinese cohort (14). We also found evidence for selection of the V10I substitution in genotype D however, in our assays a reduction in the CD8 T cell response was only detectable in combination with an additional substitution at position 4. Other studies specifically analyzing the impact of sequence variants in the core_18-27_ region on the CD8^+^ T cell response by Bertoletti et al. were consistent with immune escape by common variants (8, 10, 18). In line with our data, the authors found that the F7Y substitution did not impair HLA-A*02:01 binding and observed a similar pattern of reactivity (18). Previous work showed that epitope core 10-27 can be also presented by HLA-class II (18), its therefore possible that this region is also under HLA-class II selection pressure, which was not addressed in our study.

Different mechanisms for functional impairment of the CD8^+^ T cell response by selected substitutions in targeted epitopes have been described (31). Here, the degree of cross-reactivity of core_18-27_-specific CD8 T cells with epitope variants suggests that binding to the HLA class I-molecule is not impaired in most cases. In line with earlier studies, our results rather suggest that the interaction of the TCR with some of the variant peptides in complex with the HLA molecule is altered, which can present as an antagonistic effect of the variant peptide on T cell function (10). Importantly, impairment of TCR binding was seemingly only achieved when two substitutions within the epitope were selected. In contrast, the immunological assays were less conclusive for the single F7Y substitution in the context of HLA-A*02 and S4T substitution in the context of HLA-B*35:01/03, despite there was strong statistical support for selection in the presence of these alleles. This was in line with previous studies by Bertoletti et al. showing the ability of core_18-27_ specific CD8 T cells to tolerate substitutions at epitope positions 4-7 (18). Lack of a functional impact on the CD8 T cell response by putative escape mutations in the targeted epitope was previously described in HCV in the chimpanzee model and in the human system (26, 32). In these cases, evidence for functional immune escape was obtained from experiments, in which the endogenously processed epitope was studied. Similar to escape mutations in HCV (28), the F7Y substitution did not show evidence for immune escape in assays when the synthetic peptide was exogenously added, however, the immune response to the endogenously processed epitope was impaired by the substitution. Most likely, this is explained by reduced amounts of variant peptide antigen on the surface of cells as a consequence of altered antigen processing. Although we do not know at what extent a reduction of the T cell response is biologically relevant, we can speculate that cells infected with viral variants have less antigen on their surface and are therefore less susceptible to T cells. Unfortunately, HepG2 cells do not express HLA-B*35:01/03 and therefore the impact of the S4T substitution could not be addressed with the same assay.

The viral sequence data suggest that different pathways lead the virus to escape from the CD8^+^ T cell response. In HIV-1, of all the HLA-associated polymorphisms that occur within or near optimally described CTL epitopes, roughly 20% occur at anchor positions for HLA binding (33). The remaining substitutions are selected in other positions of the epitope and either impair antigen processing or TCR binding. Notably, there are also examples for selection of distinct escape variants within an identical epitope depending on the presenting HLA class I subtype (34, 35). The overall selection process is likely influenced by viral factors such as the impact of individual substitutions on the replication capacity as well as host factors such as the presenting HLA class molecule. Indeed, our data also suggest that different escape pathways are selected in the context of HLA-B*35:01/03 compared to the context of HLA-A*02:01. In HLA-B*35:01/03 positive patients a highly reproducible S4T substitution is selected whereas in HLA-A*02 positive patients this substitutions is rarely selected and even undetectable at the quasispecies level. In HLA-A*02, the escape pathways seem more complex and include substitutions in different positions. Multiple sites under selection pressure may suggest either continuous evolution if no optimal escape mutation can be selected or different solutions for optimal immune escape. In turn, the barrier to escape may be lower for HLA-B35:01/03. Lower expression levels of HLA-B*35 compared to other HLA class I-molecules have been reported (36) which may be consistent with a lower threshold to full escape. Distinct mutational escape pathways depending on the presenting HLA-class I-molecule may contribute to differential viral evolution in HLA-diverse populations (37, 38).

Multiple sites under selection pressure in the context of the same HLA class I-subtype may also reflect the highly individualized pathways to immune escape. Given the enormous diversity of the TCR it seems plausible that the selection process also reflects the predominant TCRs recruited by the individual’s CD8^+^ T cell response. Although we could not find clear evidence for functional immune escape even for some of the selected autologous variants in patients with chronic infection, we believe that the individual TCR plays an important role (39). Indeed, the analysis of the cross-reactivity profiles of TCR-clones indicated differences between the responses of individual TCRs to variations of the epitope. In case different epitope variants are equally well processed and presented on the cell surface, individual broadly cross-reactive TCRs will likely be advantageous compared to TCRs with a narrowly focused cross-reactivity profile. In the setting of infections with highly variable pathogens such as HBV, TCRs with broad cross-reactivity - such as clone 6K in our analysis – will therefore be quite valuable for CD8^+^ T cell-based therapies.

It is well established that HBeAg positive HBV infection associates with higher viral loads compared to HBeAg negative infection (40). Strikingly, we did not observe any variation from the prototype sequence in HBeAg positive patients, which is in line with absence of detectable core-specific CD8 T cell responses (30). In HBeAg negative patients the frequency of variants was enriched and the presence of variants was associated with stronger CD8 T cell responses compared to patients with prototype sequence. Whether this truly reflects a greater magnitude of the response or a better capacity of specific CD8 T cells to expand, needs to be studied. In HCV infection differentiation towards a memory phenotype after mutational escape has been described (41), however, this has not been observed in the context of HBV (24). In line with the idea that the immune response is an important factor for the viral replication level, we could not identify differences in the viral load associated with individual substitutions in HBeAg negative patients. However, we cannot exclude that lower viral loads are also caused by fitness costs. To directly analyze the influence of individual substitutions on viral fitness, a robust replication model for HBV is required. It has yet to be determined whether the recently developed *in vitro* models for HBV replication are sufficiently robust to address this in the future (16, 42)

Taken together, our data show that the dominant epitope region core_18-27_ is highly variable and under heavy selection pressure in the context of different HLA class I-molecules. The selection process is rather complex and may be driven by host factors such as the presenting HLA-molecule and the TCR recruited for CD8^+^ T cell response. Mechanistically, the escape pathways seem to impair binding of the TCR to the variant peptide/HLA class I-complex, which suggests that broadly cross-reactive TCRs may be beneficial in this context. However, we also find evidence for impaired antigen processing and reduced presentation of epitope variants. Viruses carrying such escape substitutions will be less susceptible to CD8^+^ T cell responses and viral genome sequencing should be considered when strategies for T cell therapies are further developed.

## Data availability statement

The datasets presented in this study can be found in online repositories. The names of the repository/repositories and accession number(s) can be found below: https://www.ncbi.nlm.nih.gov/genbank/, MZ043025-MZ043097, MZ097624-MZ097884.

## Ethics statement

The studies involving human participants were reviewed and approved by Ethikkommission an der Medizinischen Fakultät der Heinrich-Heine-Universität Düsseldorf. The patients/participants provided their written informed consent to participate in this study.

## Author contributions

The project was conceived by AW, JB, TS, and JT. Experiments were performed by AW, TS, JB, KW, CM, EA, HK, AK, and FH. Data were analyzed by all authors. The manuscript was written by AW and JT with input from all authors. All authors contributed to the article and approved the submitted version.

## Funding

This study was funded by grants from the DFG (TI 323/4–1), the Stiftung zur Erforschung infektiös-immunologischer Erkrankungen (AW, 10-16-72), the Jürgen Manchot Foundation and the European Union's Horizon 2020 research and innovation program (no. 848223; TherVacB consortium). The funders had no role in study design, data collection and interpretation, or the decision to submit the work for publication.

## Acknowledgments

The authors are very grateful to the study participants for taking part in the study. The authors thank Didier Trono, EPFL Lausanne for plasmids and protocols and Alexandra Graupner, Anja Voges and Eugen Bäcker for technical help. 

## Conflict of interest

The authors declare that the research was conducted in the absence of any commercial or financial relationships that could be construed as a potential conflict of interest.

## Publisher’s note

All claims expressed in this article are solely those of the authors and do not necessarily represent those of their affiliated organizations, or those of the publisher, the editors and the reviewers. Any product that may be evaluated in this article, or claim that may be made by its manufacturer, is not guaranteed or endorsed by the publisher.
