# Supplementary material for: Immune escape pathways from the HBV core18-27 CD8 T cell response are driven by individual HLA class I alleles
Source: Front Immunol. 2022 Nov 10;13:1045498. doi: 10.3389/fimmu.2022.1045498 (PMC9686862; doi:10.3389/fimmu.2022.1045498)
Supplement: Supplementary file 1 [file DataSheet_1.pdf]

patient-ID	core-specific IFN-g positive CD8 T cells (in %)	F	L	P	S	D	F	F	P	S	V	GENDER	HLA-A1	HLA-A2	HLA-B1	HLA-B2	Genotyp	HBV DNA [IU/ml] at time of PBMC	HBsAg	HBeAg	anti-HBe	anti-HBs	anti-HBc	Anti-HCV	HDV coinfektion	AST	ALT	Billirubin	INR	Albumin	Therapy
patient-001	0,71	nd	nd	nd	nd	nd	nd	nd	nd	nd	nd	m	0101	0301	1302	3501	D	49	4	neg	pos	neg	pos	neg	neg	20	34	0,73	1,1	4,6	No
patient-002	0	nd	nd	nd	nd	nd	nd	nd	nd	nd	nd	m	0201	2402	5101	5201	D	93	187	neg	pos	neg	pos	neg	neg	17	14	0,31	1	4,2	No
patient-003	0	nd	nd	nd	nd	nd	nd	nd	nd	nd	nd	w	0201	2901/02	0705/06	5701	A	8690	20914	neg	nd	neg	pos	neg	neg	30	39	0,42	1	4,3	No
patient-004	0,086	nd	nd	nd	nd	nd	nd	nd	nd	nd	nd	m	0201	1101	5001	5801	D	<20	12	neg	nd	neg	pos	neg	neg	29	39	0,26	0,9	4,4	nd
patient-005	0,33	nd	nd	nd	nd	nd	nd	nd	nd	nd	nd	w	0201	0301	3701	5201	D	441	865	neg	nd	neg	pos	neg	neg	18	18	0,41	1	3,8	No
patient-006	0	nd	nd	nd	nd	nd	nd	nd	nd	nd	nd	m	0201	2601	0702	4402	D	<20	3320	neg	pos	neg	pos	neg	neg	25	23	0,57	1	4,2	Yes
patient-007	0	-	-	-	-	-	-	-	-	-	-	m	2402	2402	1402	5101	D	<357	246	neg	pos	neg	pos	neg	neg	31	26	0,6	nd	4,5	nd
patient-008	0,351	-	-	-	-	-	-	-	-	-	-	w	2301	2301	5101	5101	D	<357	7522	neg	pos	neg	pos	neg	neg	17	16	0,9	1,04	4,3	nd
patient-009	0,12	-	-	-	-	-	-	-	-	-	-	m	0206	6801	0801	5501	D	nd	nd	nd	nd	nd	nd	nd	nd	nd	nd	nd	nd	nd	No
patient-010	0,081	-	-	-	-	-	-	-	-	N	I	m	0101	0201	0801	5601	A	<357	6416	neg	pos	neg	pos	neg	neg	21	35	0,1	1	4,2	nd
patient-011	0,093	nd	nd	nd	nd	nd	nd	nd	nd	nd	nd	m	3301	6802	1402	3508	D	22390	24065	neg	pos	neg	pos	neg	neg	21	28	0,4	1,01	4,2	No
patient-012	0,043	-	-	-	-	-	-	-	-	-	-	m	0301	3002	0702	0801	D	<357	364	neg	pos	neg	pos	neg	neg	24	25	0,6	1,02	4,2	nd
patient-013	1,97	-	-	-	-	-	-	-	-	-	-	m	0205	3301	3501	5801	D	<357	10237	neg	pos	neg	pos	neg	neg	28	52	1,5	1,1	4,4	nd
patient-014	1,74	-	-	-	A	-	-	-	-	-	T	m	0206	1101	0801	5108	A	1509	453	neg	pos	neg	pos	neg	neg	18	29	0,5	0,98	4,2	No
patient-015	0,37	nd	nd	nd	nd	nd	nd	nd	nd	nd	nd	m	0101	1101	4901	5101	D	<357	8089	pos	neg	neg	pos	neg	neg	49	68	0,6	1,08	4,3	Yes
patient-016	0	-	-	-	N	-	-	-	-	-	-	m	0205	0302	4901	5101	D	6027	4695	neg	pos	neg	pos	neg	neg	15	25	0,5	1,08	4,6	No
patient-017	0	nd	nd	nd	nd	nd	nd	nd	nd	nd	nd	m	2402	2402	2702	5108	D	<357	3210	pos	neg	neg	pos	neg	neg	14	25	0,6	1,01	4,6	Yes
patient-018	0	-	-	-	-	-	-	-	-	-	-	m	0301	0301	1302	3508	D	<357	5974	neg	pos	neg	pos	neg	neg	15	19	1,2	1,02	4,7	nd
patient-019	0,701	-	-	-	V	-	-	-	-	-	-	m	0201	2601	0702	0702	D	<357	206	neg	pos	neg	pos	neg	neg	23	46	0,4	nd	nd	nd
patient-020	0,037	-	-	-	-	-	-	-	-	-	-	m	0101	3101	3501	3701	D	2036	49666	pos	neg	neg	pos	neg	neg	27	36	11,5	0,95	4,7	No
patient-021	0,408	-	-	-	P	-	-	-	-	-	-	m	1101	2501	1801	5601	D	<357	1957	neg	neg	neg	pos	neg	neg	30	48	1,8	1,13	4,9	nd
patient-022	0	-	-	-	-	-	-	-	-	-	A	m	2301	2301	3501	4501	D	<357	18144	neg	pos	neg	pos	neg	neg	31	47	0,6	1,09	4,1	nd
patient-023	1,959	-	-	-	A	-	-	-	-	-	-	m	0301	6601	3503	4102	D	<357	34041	neg	pos	neg	pos	neg	neg	17	38	0,5	0,96	4,6	nd
patient-024	0	-	-	-	-	-	-	-	-	-	-	w	0201	3001	3501	5301	D	<357	1006	pos	neg	neg	pos	neg	neg	49	72	0,9	1,16	4,6	Yes
patient-025	0,486	-	-	-	I	-	-	Y	-	-	-	w	0101	0201	4402	5701	A	<357	2924	neg	pos	neg	pos	neg	neg	14	22	0,5	0,96	4,6	nd
patient-026	1,12	-	-	-	-	-	-	-	-	-	-	m	0101	0201	4901	5101	D	1528	1613	neg	pos	neg	pos	neg	neg	14	23	1,4	nd	4,7	No
patient-027	0	-	-	-	-	-	-	-	-	-	-	m	0301	2301	3501	5001	D	<357	736	neg	pos	neg	pos	neg	neg	13	23	0,5	0,99	4,3	nd
patient-028	1,69	nd	nd	nd	nd	nd	nd	nd	nd	nd	nd	m	0201	0201	0702	5101	A	<357	22621	neg	pos	neg	pos	neg	neg	25	28	0,3	1,03	4,4	Yes
patient-029	0	nd	nd	nd	nd	nd	nd	nd	nd	nd	nd	w	1102	3301	4001	5102	D	<357	83	pos	neg	neg	pos	neg	neg	20	21	0,7	0,96	4,6	Yes
patient-030	0	-	-	-	-	-	-	-	-	-	-	m	0201	2901	4501	5101	D	<357	11671	neg	pos	neg	pos	neg	neg	26	39	0,8	1,11	4,3	nd
patient-031	0,398	nd	nd	nd	nd	nd	nd	nd	nd	nd	nd	m	0201	6801	1801	4402	D	188860	3486	neg	pos	neg	pos	neg	neg	50	112	0,5	0,97	5,3	Yes
patient-032	0,03	-	-	-	-	-	-	-	-	-	-	m	2301	2402	0702	4901	D	<357	7636	neg	pos	neg	pos	neg	neg	15	22	0,7	1	4,3	nd
patient-033	0,172	-	-	-	H	-	-	Y	-	-	-	m	0101	0201	0801	2703	A	<357	10196	neg	pos	neg	pos	neg	neg	56	126	0,4	1,06	4,5	nd
patient-034	4,66	-	-	-	E	-	-	-	-	-	-	m	2602	3301	1402	5101	D	7293000	14318	neg	pos	neg	pos	neg	neg	70	152	0,4	1,05	4	No
patient-035	0,011	-	-	-	-	-	-	-	-	-	-	m	3101	3101	3501	3501	D	<357	18771	pos	neg	neg	pos	neg	neg	27	42	0,6	1,01	4,6	Yes
patient-036	0	-	-	-	-	-	-	-	-	-	-	w	1101	1101	1801	5101	D	336900	37402	pos	neg	neg	pos	neg	neg	25	34	0,8	1,11	4,3	No
patient-037	0	-	-	-	-	-	-	-	-	-	A	w	0101	0201	3701	5101	A	<357	10113	neg	pos	neg	pos	neg	neg	20	25	0,5	0,98	4,3	nd
patient-038	14,8	-	-	-	L	-	-	-	-	-	-	w	0101	0201	0801	1501	A	4564	2640	neg	pos	neg	pos	neg	neg	24	30	0,4	1,04	4,8	No
patient-039	0,06	-	-	-	A	-	-	-	-	-	-	m	0201	2901/02	1801	4403	D	38950	900,8	neg	pos	neg	pos	neg	neg	23	37	1	1,08	4,2	No
patient-040	0	-	-	-	-	-	-	-	-	-	-	m	0201	6801	4101	5101	D	<357	4625	neg	pos	neg	pos	neg	neg	22	27	0,7	1,07	4,6	nd
patient-041	0	nd	nd	nd	nd	nd	nd	nd	nd	nd	nd	m	0201	2402	0705	5001	D	179	66631	pos	neg	neg	pos	neg	neg	20	54	0,6	1,11	4,9	Yes
patient-042	0	-	-	-	-	-	-	-	-	-	-	m	0205	3101	4402	5001	D	174	3507	neg	pos	neg	pos	neg	neg	24	39	0,6	1,04	4,7	No
patient-043	1,713	nd	nd	nd	nd	nd	nd	nd	nd	nd	nd	m	0201	0301	0702	3503	D	<10	4691	neg	pos	neg	pos	neg	neg	16	25	0,5	1,06	4,2	Yes
patient-044	0,356	-	-	-	-	-	-	-	-	-	-	m	0301	6801	0801	5701	D	<10	3972	neg	pos	neg	pos	neg	neg	12	33	0,7	1,15	4,9	nd
patient-045	0,044	nd	nd	nd	nd	nd	nd	nd	nd	nd	nd	m	0302	1101	3501	3501	D	<10	6066	pos	neg	neg	pos	neg	neg	28	67	0,7	1,1	4,6	Yes
patient-046	1	-	-	-	Q	-	-	Y	-	-	-	m	0201	2501	1801	5101	D	1221	2814	neg	pos	neg	pos	neg	neg	25	29	0,5	nd	nd	No
patient-047	0,775	-	-	-	A	-	-	Y	-	-	-	m	0201	0301	1501	1501	A	1070	793	neg	pos	neg	pos	neg	neg	20	31	0,5	0,94	nd	No
patient-048	0	nd	nd	nd	nd	nd	nd	nd	nd	nd	nd	m	0201	2402	1302	3702	D	291	9681	pos	neg	neg	pos	neg	neg	24	42	0,5	0,96	nd	Yes
patient-049	0,038	nd	nd	nd	nd	nd	nd	nd	nd	nd	nd	m	0101	0205	1517	5001	D	<10	6625	pos	neg	neg	pos	neg	neg	13	22	0,4	0,94	4,3	Yes
patient-050	3,86	nd	nd	nd	nd	nd	nd	nd	nd	nd	nd	m	0201	0201	4405	5201	A	<10	neg	neg	neg	neg	pos	neg	neg	26	20	0,7	0,93	nd	Yes
patient-051	0,945	-	-	-	A	-	-	-	-	-	I	m	0201	0201	1801	4402	D	177	6313	neg	pos	neg	pos	neg	neg	12	15	0,3	1,07	4,5	No
patient-052	0	-	-	-	-	-	-	-	-	-	-	m	0301	3201	3501	3508	D	15	11698	neg	pos	neg	pos	neg	neg	23	46	0,5	1,04	4,7	Yes
patient-053	0,327	-	-	-	-	-	-	-	-	-	-	m	0201	2402	4002	4901	D	15	6512	neg	pos	neg	pos	neg	neg	20	36	1,3	1	nd	No
patient-054	0	nd	nd	nd	nd	nd	nd	nd	nd	nd	nd	w	0101	0201	1801	4402	D	13	13724	pos	neg	neg	pos	neg	neg	22	37	0,5	1,03	4,9	Yes
patient-055	0,833	-	-	-	T	-	-	Y	-	-	-	m	0201	2402	5101	5501	D	455	2053	neg	pos	neg	pos	neg	neg	17	19	0,9	1,06	4,3	No
patient-056	0,26	nd	nd	nd	nd	nd	nd	nd	nd	nd	nd	m	0101	0205	1517	5001	D	<10	3941	pos	neg	neg	pos	neg	neg	23	37	0,2	0,99	nd	Yes
patient-057	0	-	-	-	-	-	-	-	-	-	-	m	1101	6601	3501	4102	D	<10	78,7	neg	pos	neg	pos	neg	neg	21	32	1,1	1,08	nd	nd
patient-058	0,538	-	-	-	-	-	-	Y	-	-	-	m	0204	0301	4101	5701	D	885	8716	neg	pos	neg	pos	neg	neg	27	37	0,8	1,09	4,4	No
patient-059	2,643	nd	nd	nd	nd	nd	nd	nd	nd	nd	nd	w	0201	3301	1402	3901	A	11	1212	neg	pos	neg	pos	neg	neg	21	26	0,2	1,02	nd	No
patient-060	0,945	-	-	-	G	-	-	-	-	-	-	m	0301	3101	3503	5101	D	<10	155	neg	pos	neg	pos	neg	neg	26	31	0,9	0,96	4,2	nd
patient-061	0,56	-	-	-	-	-	-	Y	-	-	-	w	2601	3401	1521	5502	A	<10	1044	neg	pos	neg	pos	neg	neg	23	23	0,5	0,92	4,7	nd
patient-062	3,076	-	-	-	-	-	-	-	-	-	A	w	0201	0301	0702	1302	A	6684986	26120,5	neg	pos	neg	pos	neg	neg	739	567	24,7	1,16	2,5	No
patient-063	0	-	-	-	-	-	-	Y	-	-	-	m	0101	0204	1517	5501	D	<10	2428	neg	pos	neg	pos	neg	neg	29	29	0,4	0,9	4,4	nd
patient-064	3,134	-	-	-	Q	-	-	-	-	-	-	w	0201	2601	1501	5101	D	<10	1344	neg	pos	neg	pos	neg	neg	19	20	0,7	0,98	4,1	nd
patient-065	0	-	-	-	N	-	-	-	-	-	-	m	0101	2402	0801	3502	D	12	1747	neg	pos	neg	pos	neg	neg	28	58	0,7	1,11	4,5	No
patient-066	2,66	-	-	-	-	-	-	-	-	-	I	w	0101	0205	1517	5001	D	<10	1172	neg	pos	neg	pos	neg	neg	14	17	0,3	0,94	4,3	nd
patient-067	0	-	-	-	-	-	-	-	-	-	-	m	0101	0201	3701	5101	D	17	23525	pos	neg	neg	pos	neg	neg	40	84	0,6	1,01	4,7	No
patient-068	0	-	-	-	-	-	-	-	-	-	-	m	0301	6801	0702	3502	D	6619	1695	neg	pos	neg	pos	neg	neg	21	37	0,7	nd	nd	No
patient-069	0,167	-	-	-	-	-	-	-	-	-	-	m	0101	0301	0702	0801	A	168	5630	neg	pos	neg	pos	neg	neg	36	36	0,4	0,94	nd	No
patient-070	4,026	-	-	-	A	-	-	-	-	-	-	m	0201	3001	1302	5107	D	1866	118	neg	pos	neg	pos	neg	neg	21	51	0,6	nd	nd	No
patient-071	0	-	-	-	-	-	-	-	-	-	-	w	1101	2402	1501	3501	D	<10	1787	neg	pos	neg	pos	neg	neg	27	79	0,5	0,94	4,2	nd
patient-072	1,66	nd	nd	nd	nd	nd	nd	nd	nd	nd	nd	m	1101	6801	3503	3503	D	<10	971	neg	neg	neg	pos	neg	neg	19	20	1,1	0,94	nd	Yes
patient-073	5,62	-	-	-	A	-	-	-	-	-	-	w	0201	0301	0702	4002	A	1685	10055	neg	pos	neg	pos	neg	neg	19	26	0,4	0,92	4,2	No
patient-074	1,904	-	-	-	T	-	-	Y	-	-	-	m	0201	0301	3503	4601	D	<10	2800	neg	pos	neg	pos	neg	neg	22	32	0,5	1,2	nd	nd
patient-075	0	-	-	-	N	-	-	Y	-	-	-	w	0201	0201	4402	5201	A	526	5955	neg	pos	neg	pos	neg	neg	19	30	0,3	0,95	4,1	No
patient-076	0	-	-	-	A	-	-	-	-	-	-	w	0101	1101	3508	5201	D	799	1428	neg	pos	neg	pos	neg	neg	16	21	0,6	nd	nd	No
patient-077	4,53	nd	nd	nd	nd	nd	nd	nd	nd	nd	nd	m	0101	0301	1302	3501	D	<10	1356	neg	pos	neg	pos	neg	neg	16	20	0,6	1,1	4,3	Yes
patient-078	0,156	-	-	-	-	-	-	-	-	A	-	w	0201	0301	4402	5501	D	190	4369	neg	pos	neg	pos	neg	neg	18	19	0,7	0,98	4,5	No
patient-079	0,318	-	-	-	H	-	-	-	-	-	-	w	0201	3101	3501	5101	A	<10	2016	neg	pos	neg	pos	neg	neg	19	14	0,3	0,99	4,8	nd
patient-080	0	-	-	-	-	-	-	-	-	-	-	w	2601	3101	3801	5101	D	135	7221	pos	neg	neg	pos	neg	neg	31	47	1,1	0,99	4,4	Yes
patient-081	0,697	-	-	-	T	-	-	Y	-	-	-	m	0201	1101	5101	5101	D	22	1094	neg	pos	neg	pos	neg	neg	27	50	0,6	1,06	nd	No
patient-082	0	nd	nd	nd	nd	nd	nd	nd	nd	nd	nd	m	0201	3001	1501	5501	A	7608000	14127	pos	pos	neg	pos	neg	neg	127	298	1	1,13	nd	Yes
patient-083	0	-	-	-	-	-	-	-	-	-	A	w	0101	0201	1501	5101	D	1424	13201	neg	pos	neg	pos	neg	neg	24	18	0,7	0,97	nd	No
patient-084	6,66	-	-	-	-	-	-	-	-	-	-	m	0201	3001	1302	5107	D	171	2492	neg	pos	neg	pos	neg	neg	16	27	0,5	1	nd	No
patient-085	0,379	-	-	-	A	-	-	-	-	-	-	m	0206	2601	3503	3801	D	59	4537	neg	pos	neg	pos	neg	neg	25	36	0,5	1,04	nd	No
patient-086	0,489	-	-	-	-	-	-	-	-	-	-	w	0201	6801	1501	4402	D	37	158	neg	pos	neg	pos	neg	neg	23	26	0,2	1	nd	No
patient-087	3,38	-	-	-	-	-	-	-	-	-	-	w	3301	6801	3501	5801	A	898	14587	neg	pos	neg	pos	neg	neg	26	9	1,3	1,08	4,1	No
patient-088	9,61	-	-	-	T	-	-	-	-	-	-	w	1101	3002	0801	3503	D	<10	2709	neg	pos	neg	pos	neg	neg	25	37	0,4	0,98	4,5	nd
patient-089	7,621	-	-	-	G	-	-	-	-	-	I	m	0201	0201	3501	3503	D	<10	2243	neg	pos	neg	pos	neg	neg	21	16	0,5	0,98	4,5	nd
patient-090	0	-	-	-	-	-	-	-	-	-	-	m	3201	6801	3501	5501	D	2308	2286	neg	pos	neg	pos	neg	neg	25	21	0,8	0,98	4,5	No
patient-091	0,44	-	-	-	-	-	-	-	-	-	-	w	0203	0302	3802	5101	D	<10	1849	neg	pos	neg	pos	neg	neg	24	17	0,6	0,95	4,4	nd
patient-092	0,475	-	-	-	T	-	-	-	-	-	-	m	0101	0101	3802	5701	D	410	130	neg	pos	neg	pos	neg	neg	22	33	0,5	1,21	4,3	No
patient-093	3,248	-	-	-	-	-	-	-	-	-	-	w	0205	0217	5001	5101	D	1112	2513	neg	pos	neg	pos	pos	neg	12	16	0,9	0,94	4,4	No
patient-094	0,13	nd	nd	nd	nd	nd	nd	nd	nd	nd	nd	m	0301	2402	1501	3503	D	151	23	neg	pos	neg	pos	neg	neg	25	25	0,9	0,98	4,6	No
patient-095	0	nd	nd	nd	nd	nd	nd	nd	nd	nd	nd	m	0201	0301	3501	4002	D	<10	6	neg	neg	neg	pos	neg	neg	58	100	0,6	1,05	4,8	Yes
patient-096	0	-	-	-	-	-	-	-	-	-	-	m	2402	3001	1302	3508	D	<10	14539	neg	neg	neg	pos	neg	neg	19	38	0,5	1,02	4,4	nd
patient-097	0,093	-	-	-	-	-	-	-	-	-	-	m	2901/02	3301	5001	5101	D	23	6602	neg	pos	neg	pos	neg	neg	17	33	0,5	1,08	nd	Yes
patient-098	0,363	-	-	-	-	-	-	-	-	-	-	m	0201	2402	3501	4001	D	<10	1935	neg	pos	neg	pos	neg	neg	18	21	1	0,97	nd	nd
patient-099	0	-	-	-	-	-	-	-	-	-	-	m	2601	3101	3502	3503	D	<10	2378	neg	pos	neg	pos	neg	neg	38	30	0,8	nd	nd	nd
patient-100	0	nd	nd	nd	nd	nd	nd	nd	nd	nd	nd	m	0201	3301	1302	1402	D	<10	949	pos	neg	neg	pos	neg	neg	24	47	1	0,98	5	Yes
patient-101	0,395	nd	nd	nd	nd	nd	nd	nd	nd	nd	nd	w	0101	1101	0702	5001	A	<10	21580	neg	pos	neg	pos	neg	neg	14	22	0,6	0,93	4,7	Yes
patient-102	1,391	-	-	-	-	-	-	-	-	-	-	m	0201	0302	3503	4402	D	<10	5988	neg	pos	neg	pos	neg	neg	22	40	0,7	1,08	nd	nd
patient-103	0	-	-	-	A	-	-	-	-	-	-	w	2402	2402	0702	3502	A	<10	940	neg	pos	neg	pos	neg	neg	15	17	0,4	0,94	4,3	nd
patient-104	0,31	-	-	-	-	-	-	-	-	-	-	m	0101	3301	0801	3501	D	<10	3017	neg	neg	neg	pos	neg	neg	17	16	0,6	1,06	nd	Yes
patient-105	24,6	-	-	-	T	-	-	-	-	-	-	m	1101	3301	3503	5001	D	<10	171	neg	pos	neg	pos	nd	neg	21	38	0,7	0,94	4,5	nd
patient-106	0,186	-	-	-	A	-	-	-	-	-	-	m	0201	2402	1801	2703	D	7567	6559	neg	pos	neg	pos	neg	neg	12	32	0,6	1,05	3,9	No
patient-107	15,03	-	-	-	I	-	-	-	-	-	-	m	0201	0205	5001	5701	A	1700	3346	neg	pos	neg	pos	neg	neg	24	24	0,5	1	4,6	No
patient-108	0	-	-	-	-	-	-	-	-	-	-	m	0201	3301	1302	1402	D	6529	9518	pos	neg	neg	pos	neg	neg	28	67	0,5	1,07	nd	No
patient-109	0,368	-	-	-	-	-	-	-	-	-	-	m	0206	2402	3508	5501	D	3920	770	neg	pos	neg	pos	neg	neg	35	26	0,7	1,08	nd	No
patient-110	0,845	nd	nd	nd	nd	nd	nd	nd	nd	nd	nd	w	1101	2402	3801	5801	D	23	2431	neg	pos	neg	pos	neg	neg	22	32	0,3	0,97	4,9	No
patient-111	0	-	-	-	A	-	-	-	-	-	-	w	0101	2501	0801	3502	D	332	1597	neg	pos	neg	pos	neg	neg	13	14	0,2	0,94	3,3	No
patient-112	0,235	-	-	-	-	-	-	-	-	-	-	w	1101	3001	1302	3501	D	<10	98,2	neg	pos	neg	pos	neg	neg	24	19	1	1,1	4,6	nd
patient-113	0,029	-	-	-	-	-	-	-	-	-	-	m	0201	0201	5101	5701	A	<10	7485	neg	pos	neg	pos	neg	neg	22	24	0,4	0,89	4,6	nd
patient-114	0,092	-	-	-	-	-	-	-	-	-	-	m	2402	3001	3502	4201	D	753	728	neg	pos	neg	pos	neg	neg	21	25	0,3	0,92	4,4	No
patient-115	1,343	-	-	-	-	-	-	-	-	-	-	m	0101	2402	2702	3501	D	532	5075	neg	pos	neg	pos	neg	neg	34	41	0,7	0,98	4,6	No
patient-116	38,066	-	-	-	T	-	-	Y	-	-	-	m	0206	2601	3503	3801	D	<10	945	neg	pos	neg	pos	neg	neg	24	46	0,7	0,92	4,9	nd
patient-117	0,755	-	-	-	-	-	-	-	-	-	-	m	1101	6801	5101	5201	D	nd	2226	neg	pos	neg	pos	neg	neg	63	50	1,1	1,07	4,1	No
patient-118	0	nd	nd	nd	nd	nd	nd	nd	nd	nd	nd	w	0101	0301	0801	5101	D	356	279	neg	pos	neg	pos	neg	neg	nd	nd	nd	nd	nd	No
patient-119	5,397	-	-	-	-	-	-	-	-	-	-	m	0201	6802	4002	5301	A	<10	7420	neg	pos	neg	pos	neg	neg	38	57	0,5	1,03	4,1	nd
patient-120	0,497	-	-	-	-	-	-	-	-	-	A	w	0201	2901/02	0801	5101	D	<10	1,14	neg	neg	neg	pos	neg	neg	32	34	0,5	0,97	nd	nd
patient-121	0	-	-	-	-	-	-	-	-	-	-	w	2402	3201	3503	5101	D	1050000	17722	pos	neg	neg	pos	neg	neg	22	21	0,9	0,93	4,7	No
patient-122	0,05	-	-	-	T	-	-	-	-	-	-	w	0301	3101	1518	5101	D	<10	neg	neg	pos	neg	pos	neg	neg	17	24	0,6	0,95	4,4	nd
patient-123	0	nd	nd	nd	nd	nd	nd	nd	nd	nd	nd	w	1101	2402	1801	3501	D	<10	37,3	neg	pos	neg	pos	neg	neg	28	40	0,6	1,15	4,5	nd
patient-124	0,33	-	-	-	-	-	-	-	-	-	-	m	0101	0301	0801	3501	A	<10	2195	neg	neg	neg	pos	neg	neg	12	17	0,4	0,93	4,6	nd
patient-125	0	nd	nd	nd	nd	nd	nd	nd	nd	nd	nd	w	0201	0201	3503	4601	D	<10	2,55	neg	pos	neg	pos	neg	neg	27	23	0,6	0,94	4,5	Yes
patient-126	0,105	nd	nd	nd	nd	nd	nd	nd	nd	nd	nd	w	0101	3101	5101	5801	D	51	27537	neg	pos	neg	pos	neg	neg	33	90	0,4	0,97	4,5	No
patient-127	0	nd	nd	nd	nd	nd	nd	nd	nd	nd	nd	m	0101	6801	0702	3508	D	<10	neg	neg	pos	neg	pos	neg	neg	39	44	0,6	1,11	4,2	Yes
patient-128	0,34	nd	nd	nd	nd	nd	nd	nd	nd	nd	nd	w	1101	6801	3501	4403	D	67	184	neg	pos	neg	pos	neg	neg	21	16	0,6	0,89	4,8	No
patient-129	0	nd	nd	nd	nd	nd	nd	nd	nd	nd	nd	m	2601	3201	3501	5101	D	70	11809	neg	pos	neg	pos	neg	neg	20	46	0,9	1,05	4,5	Yes
patient-130	0,115	nd	nd	nd	nd	nd	nd	nd	nd	nd	nd	w	2601	6801	3501	5101	D	<10	35,9	neg	pos	neg	pos	neg	neg	16	23	0,9	0,96	4,8	Yes
patient-131	2,219	nd	nd	nd	nd	nd	nd	nd	nd	nd	nd	m	6802	6901	3501	4901	D	<10	38,4	neg	pos	neg	pos	neg	neg	24	45	0,5	nd	nd	Yes
patient-132	2,689	-	-	-	T	-	-	-	-	-	-	w	0301	2402	1801	3503	D	<10	2564	neg	pos	neg	pos	neg	neg	17	20	0,3	0,91	4,4	nd
patient-133	0	nd	nd	nd	nd	nd	nd	nd	nd	nd	nd	m	0201	0201	4402	4402	A	71740	4783,7	neg	pos	nd	nd	nd	nd	nd	nd	nd	nd	nd	No
patient-134	0,173	-	-	-	T	-	-	-	-	-	-	m	0101	3201	3501	5101	D	184	1,98	neg	pos	neg	pos	neg	neg	80	104	0,3	1,03	4,6	No
patient-135	1,36	nd	nd	nd	nd	nd	nd	nd	nd	nd	nd	m	0201	1101	5101	5201	D	<10	neg	neg	pos	neg	pos	neg	neg	28	56	0,5	1,01	4,4	Yes
patient-136	7,626	-	-	-	A	-	-	-	-	-	-	m	0201	2601	2703	3501	D	228	87	neg	pos	neg	pos	neg	neg	18	25	0,6	nd	nd	No
patient-137	0	-	-	-	-	-	-	-	-	-	-	m	0201	6801	1301	5101	D	3810000	7479	pos	neg	neg	pos	neg	neg	33	49	0,4	1,05	4,1	No
patient-138	0	nd	nd	nd	nd	nd	nd	nd	nd	nd	nd	w	0101	2301	3502	4403	D	44	138	neg	pos	neg	pos	neg	neg	20	21	0,9	nd	nd	Yes
patient-139	1,785	-	-	-	Q	-	-	-	-	N	-	w	0201	0302	3503	4402	D	<10	1,82	neg	pos	neg	pos	neg	neg	25	32	0,2	0,88	4,2	nd
patient-140	7,518	-	-	-	-	-	-	-	-	-	-	w	0201	2402	1501	4402	A	<10	17666	neg	pos	neg	pos	neg	neg	21	23	0,3	0,86	4,2	Yes
patient-141	0	-	-	-	-	-	-	-	-	-	-	m	0201	0301	1302	3508	D	11	9095	pos	neg	neg	pos	neg	neg	25	60	1,3	1,03	3,9	Yes
patient-142	0,18	nd	nd	nd	nd	nd	nd	nd	nd	nd	nd	w	0101	0201	0801	4001	D	29	2717	neg	pos	neg	pos	neg	neg	25	20	0,5	0,98	4,5	No
patient-143	0	-	-	-	-	-	-	-	-	-	-	m	1101	2601	3501	3801	D	3873	6271	neg	pos	neg	pos	neg	neg	21	38	0,6	0,95	4,3	No
patient-144	0,47	nd	nd	nd	nd	nd	nd	nd	nd	nd	nd	m	0201	3101	3501	5101	D	26	54146	neg	pos	neg	pos	nd	neg	19	27	1	nd	nd	No
patient-145	0	nd	nd	nd	nd	nd	nd	nd	nd	nd	nd	m	0201	0201	4402	4402	A	116	449	neg	pos	neg	pos	neg	neg	22	28	0,8	nd	nd	No

patient-146	0,21	nd	nd	nd	nd	nd	nd	nd	nd	nd	nd	w	0201	6801	0702	4402	D	432	753	neg	pos	neg	pos	nd	neg	30	21	0,5	nd	nd	No
patient-147	0,3	nd	nd	nd	nd	nd	nd	nd	nd	nd	nd	m	0201	3201	0801	1801	D	nd	1863	pos	pos	neg	pos	neg	neg	45	65	0,4	1,02	4,9	No
patient-148	0,36	-	-	-	-	-	-	-	-	-	-	m	0201	2301	3501	4901	D	<10	4445	neg	pos	neg	pos	neg	neg	42	47	0,9	nd	nd	Yes
patient-149	0,067	-	-	-	H	-	-	-	-	-	I	w	0201	0301	1501	1801	A	73	9177	neg	pos	neg	pos	neg	neg	21	16	0,5	1,03	4,6	No
patient-150	0	nd	nd	nd	nd	nd	nd	nd	nd	nd	nd	m	0101	0201	0702	2703	D	31	1527	neg	pos	neg	pos	neg	neg	60	51	1,1	1,07	3,9	Yes
patient-151	nd	-	-	-	-	-	-	-	-	-	-	m	30xx	28xx	13xx	27xx	D	20	nd	neg	nd	neg	pos	neg	neg	6	21	0,5	1	4,6	No
patient-152	3,59	-	-	-	-	-	-	Y	-	-	-	w	0201	2402	2703	4402	D	1918	3114	neg	nd	neg	pos	neg	neg	23	18	0,41	1,05	4	No
patient-153	nd	-	-	-	H	-	-	-	-	-	I	m	0201		0702	4402	A	4424	17599	neg	pos	neg	pos	neg	neg	28	32	0,5	0,9	4,7	No
patient-154	nd	-	-	-	I	-	-	-	-	-	-	w	0201	3101	0702	3901	A	2103	27124	neg	nd	neg	pos	neg	neg	33	22	0,58	1	4,3	No
patient-155	nd	-	-	-	-	-	-	-	-	-	-	w	0201	2901/02	0702	5701	A	13863	29523	neg	nd	neg	pos	neg	neg	25	28	0,48	1	4,6	No
patient-156	nd	-	-	-	-	-	-	-	-	-	-	m	0201	6601	0702	4102	A	961	6288,48	neg	nd	neg	pos	neg	neg	28	29	nd	1,08	4,6	No
patient-157	nd	-	-	-	H	-	-	Y	-	-	-	m	0201	0235	0702	4427	A	845	7091,91	neg	nd	neg	pos	neg	neg	60	50	1	1,05	5,3	No
patient-158	nd	-	-	-	H	-	-	Y	-	-	-	m	0201	0206	0702	0801	A	88553	117956	neg	nd	neg	pos	neg	neg	35	65	nd	nd	nd	No
patient-159	nd	-	-	-	-	-	-	-	-	-	-	w	0103	6601	0702	4102	A	2288	nd	neg	nd	neg	pos	neg	neg	nd	23	5	nd	nd	No
patient-160	nd	-	-	-	H	-	-	Y	-	-	-	m	0201	0301	0702		A	14125	nd	neg	nd	neg	pos	neg	neg	nd	40	11	nd	nd	No
patient-161	nd	-	-	-	-	-	-	-	-	-	-	w	0301	1101	0702	4402	A	17378	nd	neg	nd	neg	pos	neg	neg	nd	26	11	nd	nd	No
patient-162	nd	-	-	-	-	-	-	-	-	-	-	w	0101	2601	0702	4402	D	28734	28734	neg	pos	neg	pos	neg	neg	21	20	0,39	1	4,4	No
patient-163	nd	-	-	-	-	-	-	-	-	-	-	w	0101		0702	1302	D	1350	621	neg	nd	neg	pos	neg	neg	28	16	0,54	1,1	4,8	No
patient-164	nd	-	-	-	-	-	-	-	-	-	-	w	0301	3101	0702	1518	D	7000000	61895	pos	neg	neg	pos	neg	neg	22	16	0,29	1,1	4,5	Yes
patient-165	nd	-	-	-	-	-	-	-	-	-	-	m	0101	2601	0702		D	24543	2532	neg	pos	neg	pos	neg	neg	33	41	0,5	0,9	4,7	No
patient-166	0,294	-	-	-	H	-	-	-	-	-	I	m	0201	2601	0702	4402	D	1828	91	neg	nd	neg	pos	neg	neg	39	38	0,7	1,1	4,5	No
patient-167	nd	-	-	-	A	-	-	Y	-	-	-	m	0201		0702	3501	D	17544	1666	neg	nd	neg	pos	neg	neg	25	30	0,97	1	4,4	No
patient-168	nd	-	-	-	-	-	-	-	-	-	-	m	1101	2403	0702	3502	D	5543199	19059	neg	pos	neg	pos	neg	neg	29	35	0,32	1,1	4,1	No
patient-169	nd	-	-	-	A	-	-	-	-	-	-	w	0201	0301	0702		D	3215	4947	neg	nd	neg	pos	neg	neg	24	26	0,36	1	4,2	No
patient-170	nd	-	-	-	-	-	-	-	-	-	-	m	0301	3101	0702	4801	D	102	33	neg	nd	neg	pos	neg	neg	23	35	0,23	1	4,3	No
patient-171	nd	-	-	-	A/V	-	-	-	-	-	-	w	0201		0702	3501	D	1274	6506,61	neg	nd	neg	pos	neg	neg	47	53	0,7	1	4,9	No
patient-172	nd	-	-	-	-	-	-	-	-	-	-	w	0201		0702	1501	D	6961546	nd	pos	nd	neg	pos	neg	neg	77	55	0,7	1,01	4,2	No
patient-173	nd	-	-	-	-	-	-	-	-	-	-	m	0301	3303	0702	3501	D	134344	nd	pos	nd	neg	pos	neg	neg	54	65	nd	1	4,5	Yes
patient-174	nd	-	-	-	-	-	-	-	-	-	-	w	0201	2301	0702	4403	D	2974	nd	neg	nd	neg	pos	neg	neg	nd	91	0,2	1,09	4,6	No
patient-175	nd	-	-	-	T	-	-	-	-	-	L	w	2601	3101	0702	5101	D	1918	nd	neg	nd	neg	pos	neg	neg	35	52	0,3	1,02	4,6	No
patient-176	nd	-	-	-	A	-	-	-	-	-	I	m	0101	0201	0702	5701	D	12251	nd	neg	nd	neg	pos	neg	neg	30	19	0,3	0,87	4,5	No
patient-177	nd	-	-	-	-	-	-	-	-	-	-	w	0201	1101	0702	4405	D	851	nd	neg	nd	neg	pos	neg	neg	24	20	nd	nd	5	No
patient-178	nd	-	-	-	A	-	-	Y	-	-	-	w	0201	2402	0702	4403	D	13524	nd	neg	nd	neg	pos	neg	neg	26	20	0,2	1	4,4	No
patient-179	nd	-	-	-	-	-	-	-	-	-	-	w	0301	2501	0702	5701	D	24545	nd	neg	nd	neg	pos	neg	neg	nd	23	nd	0,88	nd	No
patient-180	nd	-	-	-	-	-	-	-	-	-	-	m	0101	0201	0702	1801	D	29455901	18577,07	neg	nd	neg	pos	neg	neg	65	140	0,3	1,27	4,2	No
patient-181	nd	-	-	-	-	-	-	-	-	-	-	m	0201	2402	0702	1801	D	145426465	nd	pos	nd	neg	pos	neg	neg	127	324,3	2,9	1,08	nd	Yes
patient-182	nd	-	-	-	-	-	-	-	-	-	-	nd	0101	0201	0702	5701	D	139000000	25195	pos	nd	neg	pos	neg	neg	132	253	nd	nd	nd	Yes
patient-183	nd	-	-	-	-	-	-	-	-	-	-	nd	0101	0301	0702	1402	D	7550	9934	neg	nd	neg	pos	neg	neg	56	85	nd	nd	nd	Yes
patient-184	nd	-	-	-	A	-	-	-	-	-	-	m	0201	3201	0702	3508	D	721	166	neg	nd	neg	pos	neg	neg	nd	21	7	nd	nd	No
patient-185	nd	-	-	-	T	-	-	-	-	-	-	m	2301	3101	0702	3503	D	513	250	neg	nd	neg	pos	neg	neg	nd	24	5	nd	nd	No
patient-186	nd	-	-	-	-	-	-	-	-	-	I	w	0101	0301	0702	5701	D	1380	nd	neg	nd	neg	pos	neg	neg	nd	27	6	nd	nd	No
patient-187	nd	-	-	-	-	-	-	-	-	-	-	w	0101	1101	0705	5001	A	5660	5278	neg	nd	neg	pos	neg	neg	26	29	nd	nd	nd	No
patient-188	nd	-	-	-	-	-	-	-	-	-	-	w	0301		0705		A	14142	13716	neg	nd	neg	pos	neg	neg	33	32	nd	nd	nd	No
patient-189	nd	-	-	-	G	-	-	-	-	-	-	m	2901	6801	0705	5101	D	1290	6498,29	neg	nd	neg	pos	neg	neg	29	29	1,1	1	4,5	No
patient-190	nd	-	-	-	-	-	-	-	-	-	-	m	3101	3402	0705	1402	D	35631	2178	neg	nd	neg	pos	neg	neg	nd	49	7	nd	nd	No
patient-191	nd	-	-	-	H	-	-	Y	-	-	-	w	0101	0201	0801	3901	A	5442	22966	neg	pos	neg	pos	neg	neg	20	17	0,37	1	4,5	No
patient-192	nd	-	-	-	-	-	-	-	-	-	-	W	0301	2402	0801	5001	A	173	298	neg	nd	neg	pos	neg	neg	24	28	0,43	1	4,7	No
patient-193	nd	-	-	-	T	-	-	-	-	-	-	m	0101	2601	0801	3501	A	283,6	3447,09	neg	nd	neg	pos	neg	neg	38	52	0,5	0,91	4,7	No
patient-194	nd	-	-	-	I	-	-	-	-	-	-	m	0205	2501	0801	5001	A	2538,8	7398,42	neg	nd	neg	pos	neg	neg	36	52	0,6	0,97	4,3	No
patient-195	nd	-	-	-	H	-	-	Y	-	-	-	m	0101	0201	0801	5201	A	566	6069,24	neg	nd	neg	pos	neg	neg	27	28	0,3	0,99	4,7	No
patient-196	nd	-	-	-	-	-	-	-	-	-	-	m	0101	2402	0801		A	515	6682,83	neg	nd	neg	pos	neg	neg	34	25	nd	1,02	nd	No
patient-197	nd	-	-	-	H	-	-	-	-	-	-	w	0101	3301	0801	1402	A	1004500	6326,95	neg	nd	neg	pos	neg	neg	71	64	0,3	0,93	4,4	No
patient-198	nd	-	-	-	-	-	-	-	-	-	-	m	0101	1101	0801	4402	A	194789	6962	neg	nd	neg	pos	neg	neg	32	48	nd	nd	nd	No
patient-199	nd	-	-	-	A	-	-	-	-	-	-	w	0101	3201	0801		A	2955	25227	neg	nd	neg	pos	neg	neg	32	57	nd	nd	nd	Yes
patient-200	nd	-	-	-	A	-	-	-	-	-	A	w	2402	2501	0801	1801	A	1397	nd	neg	nd	neg	pos	neg	neg	nd	27	5	nd	nd	No
patient-201	nd	-	-	-	-	-	-	-	-	-	-	m	0101	0301	0801	1801	D	504807	7275	neg	pos	neg	pos	neg	neg	88	192	0,58	1,1	4,2	No
patient-202	nd	-	-	-	-	-	-	-	-	-	-	w	0101	1101	0801	1801	D	2008	258	neg	nd	neg	pos	neg	neg	21	14	0,41	1,1	4,5	No
patient-203	nd	-	-	-	V	-	-	-	-	-	-	m	2xx	2601	0801	5108	D	461	10891	neg	nd	neg	pos	neg	neg	32	51	1,63	1	4,7	No
patient-204	nd	-	-	-	-	-	-	-	-	-	-	m	2601		0801	3503	D	1705	8597	neg	nd	neg	pos	neg	neg	36	64	0,89	1,1	nd	No
patient-205	nd	-	-	-	A	-	-	-	-	-	-	m	2301	2601	0801	4901	D	472	461	neg	pos	neg	pos	neg	neg	39	83	0,4	1	4,3	Yes
patient-206	nd	-	-	-	T	-	-	-	-	-	-	w	0101	0301	0801	3503	D	6500	208	neg	nd	neg	pos	neg	neg	20	22	0,99	1	3,7	No
patient-207	nd	-	-	-	-	-	-	-	-	-	-	w	0101	2501	0801	1801	D	505	nd	neg	nd	neg	pos	neg	neg	21	19	nd	nd	nd	No
patient-208	nd	-	-	-	-	-	-	-	-	-	-	m	0201		0801	5101	D	400	7072	neg	nd	neg	pos	neg	neg	52	52	0,6	1,1	4,2	Yes
patient-209	nd	-	-	-	-	-	-	-	-	-	-	m	0101	0201	0801	2705	D	1011579	nd	neg	nd	neg	pos	neg	neg	42	50	nd	1,19	nd	Yes
patient-210	nd	-	-	-	-	-	-	-	-	-	-	w	0201	2402	0801	1801	D	1748	nd	neg	nd	neg	pos	neg	neg	23	12	0,5	0,95	nd	No
patient-211	nd	-	-	-	-	-	-	-	-	-	-	w	0201	3201	0801	3503	D	311	nd	neg	nd	neg	pos	neg	neg	20	18	0,6	nd	nd	No
patient-212	nd	-	-	-	-	-	-	-	-	-	-	m	0201	3001	0801	5001	D	902418212	393,33	pos	nd	neg	pos	neg	neg	43	60	0,7	1,21	4,7	No
patient-213	nd	-	-	-	-	-	-	-	-	-	-	w	0101	2402	0801	1501	D	432	nd	neg	nd	neg	pos	neg	neg	15	nd	nd	0,99	nd	No
patient-214	nd	-	-	-	A	-	-	-	-	-	-	m	0101	2301	0801	5001	D	28420670	2371,52	neg	nd	neg	pos	neg	neg	102	255	0,6	1,03	4,3	No
patient-215	nd	-	-	-	-	-	-	-	-	-	-	m	0101	1101	0801		D	355317831	46621,78	pos	nd	neg	pos	neg	neg	42	63	0,7	0,99	4,7	No
patient-216	nd	-	-	-	-	-	-	-	-	-	-	w	0101	0301	0801	5101	D	2048	nd	neg	nd	neg	pos	neg	neg	35	49	0,4	nd	nd	Yes
patient-217	nd	-	-	-	P	-	-	-	-	-	-	m	0101	2601	0801	3801	D	9236	nd	neg	nd	neg	pos	neg	neg	37	52	1,2	1,07	4,8	No
patient-218	nd	-	-	-	A	-	-	-	-	-	-	m	0101		0801		D	116	nd	neg	nd	neg	pos	neg	neg	66	64	48	0,99	nd	Yes
patient-219	nd	-	-	-	-	-	-	-	-	-	-	m	0101	3301	0801	1402	D	69100000	nd	neg	nd	neg	pos	neg	neg	1705	1605	14,9	1,4	nd	Yes
patient-220	nd	-	-	-	-	-	-	-	-	-	-	m	0101	6601	0801	4102	D	1811	nd	neg	nd	neg	pos	neg	neg	47	95	0,6	1	nd	Yes
patient-221	nd	-	-	-	V	-	-	-	-	-	-	m	0101	3201	0801		D	6956	nd	neg	nd	neg	pos	neg	neg	31	39	0,4	0,94	4,9	No
patient-222	nd	-	-	-	-	-	-	Y	-	-	-	m	0301	1101	0801	5601	D	3806	494	neg	nd	neg	pos	neg	neg	38	57	nd	nd	nd	Yes
patient-223	nd	-	-	-	-	-	-	-	-	-	-	nd	0101		0801		D	3906614	15905	neg	nd	neg	pos	neg	neg	52	128	nd	nd	nd	Yes
patient-224	nd	-	-	-	-	-	-	-	-	-	-	w	0101	0301	0801	4403	D	317100	17560	pos	nd	neg	pos	neg	neg	49	91	nd	nd	nd	Yes
patient-225	nd	-	-	-	-	-	-	-	-	-	-	m	0205	6802	1301	3910	D	5308	nd	neg	nd	neg	pos	neg	neg	nd	34	nd	1	4,6	No
patient-226	nd	-	-	-	-	-	-	-	-	-	-	w	2402	3001	1302	5101	A	5314	6280	neg	pos	neg	pos	neg	neg	17	14	nd	1	3,9	No
patient-227	nd	-	-	-	A	-	-	Y	-	-	-	w	0101	0201	1302	3501	A	489,6	4822,44	neg	nd	neg	pos	neg	neg	21	16	0,3	nd	4,7	No
patient-228	nd	-	-	-	-	-	-	-	-	-	-	m	0201	3001	1302	3901	A	220199655,8	2699,7	pos	nd	neg	pos	neg	neg	nd	79	0,5	1,05	nd	No
patient-229	nd	-	-	-	-	-	-	-	-	-	-	m	1101	3201	1302	4402	A	517	23693	neg	nd	neg	pos	neg	neg	28	29	0,7	2,25	4,3	No
patient-230	nd	-	-	-	H	-	-	-	-	-	I	w	0201	2403	1302	1801	D	25011	5496	neg	nd	neg	pos	neg	neg	15	11	0,43	1	4	No
patient-231	nd	-	-	-	-	-	-	-	-	-	-	m	0201	0301	1302	3501	D	7000000	75546	pos	neg	neg	pos	neg	neg	88	206	0,42	1,1	4,7	No
patient-232	nd	-	-	-	-	-	-	-	-	-	-	w	2402	3001	1302	4801	D	53273	nd	neg	nd	neg	pos	neg	neg	27	29	0,5	1,1	4,2	No
patient-233	nd	-	-	-	-	-	-	-	-	-	-	m	0101	2402	1302	5701	D	338,6	4921,53	neg	nd	neg	pos	neg	neg	28	24	0,3	1,02	4,7	No
patient-234	nd	-	-	-	-	-	-	-	-	-	-	m	2402	3001	1302	5101	D	1149495	nd	pos	nd	neg	pos	neg	neg	33	55	0,3	1,02	nd	No
patient-235	nd	-	-	-	A	-	-	-	-	-	-	w	0301	3201	1302	4002	D	685	nd	neg	nd	neg	pos	neg	neg	130	101	0,6	0,98	4,6	No
patient-236	nd	-	-	-	A/S	-	-	-	-	-	-	w	0201	2601	1302	3801	D	925	nd	neg	nd	neg	pos	neg	neg	22	18	0,6	0,96	4,8	No
patient-237	nd	-	-	-	-	-	-	-	-	-	-	m	1101	2301	1302	3501	D	93325	nd	neg	nd	neg	pos	neg	neg	26	39	0,2	0,95	nd	No
patient-238	nd	-	-	-	-	-	-	-	-	-	-	m	0101	3001	1302	5701	D	647540559	nd	pos	nd	neg	pos	neg	neg	28	41	0,2	nd	4,3	No
patient-239	nd	-	-	-	A	-	-	-	-	-	-	w	0201	1101	1401	1502	A	3981	nd	neg	nd	neg	pos	neg	neg	nd	34	4	nd	nd	No
patient-240	nd	-	-	-	A	-	-	-	-	-	I	m	0101	0201	1401	4402	D	1058	11031	neg	pos	neg	pos	neg	neg	22	27	0,53	1,1	4,3	No
patient-241	nd	-	-	-	P	-	-	-	-	-	A	m	0101	3301/03	1402	5101	A	1480	11828	neg	pos	neg	pos	neg	neg	28	38	0,43	1,1	4,4	Yes
patient-242	nd	-	-	-	A	-	-	-	-	-	-	m	3301/03	3601	1402	5301	A	20745	32323	neg	pos	neg	pos	neg	neg	40	64	0,89	1,1	4,7	Yes
patient-243	nd	-	-	-	G	-	Y	-	-	-	-	m	0302	3301/03	1402	5101	D	3588	3730	neg	pos	neg	pos	neg	neg	22	23	0,39	0,9	4,1	No
patient-244	nd	-	-	-	G	-	-	-	-	-	-	w	0101		1402	3501	D	25000000	6496,4	neg	nd	neg	pos	neg	neg	201	220	0,6	0,92	nd	No
patient-245	nd	-	-	-	-	-	-	-	-	-	-	m	0301	2402	1402	3502	D	64714	nd	neg	nd	neg	pos	neg	neg	36	59	0,7	1,19	4,8	Yes
patient-246	nd	-	-	-	-	-	-	-	-	-	-	w	0201	3301	1402	3503	D	401339	nd	neg	nd	neg	pos	neg	neg	nd	736	nd	1,06	nd	No
patient-247	nd	-	-	-	T	-	-	Y	-	-	-	w	0201	2501	1402	1801	D	15176	nd	neg	nd	neg	pos	neg	neg	20	29	0,4	1,03	nd	No
patient-248	nd	-	-	-	-	-	-	-	-	-	-	w	2402	3301	1402	4901	D	3871	1473,53	neg	nd	neg	pos	neg	neg	28	30	0,3	0,97	4,5	No
patient-249	nd	-	-	-	-	-	-	-	-	-	-	m	0201	2501	1402	3503	D	7322000	nd	pos	nd	neg	pos	neg	neg	90	64	1,9	1,28	3,5	No
patient-250	nd	-	-	-	-	-	-	-	-	-	-	nd	0101	3201	1402	5701	D	43164540	7883	neg	nd	neg	pos	neg	neg	150	266	nd	nd	nd	Yes
patient-251	nd	-	-	-	-	-	-	-	-	-	-	m	2403	3301/03	1402	5001	D	1953	1129	neg	nd	neg	pos	neg	neg	47	148	nd	nd	nd	No
patient-252	nd	-	-	-	H	-	-	-	-	-	-	w	0201	0301	1501	3503	A	557	3230	neg	nd	neg	pos	neg	neg	32	27	0,29	1	4,1	No
patient-253	nd	-	-	-	A	-	-	-	-	-	-	m	0101	0301	1501	5701	A	11700	23377	neg	nd	neg	pos	neg	neg	27	47	nd	nd	nd	No
patient-254	nd	-	-	-	H	-	-	-	-	-	-	w	0201	6801	1501	1801	D	3911	5307	neg	pos	neg	pos	neg	neg	19	13	0,28	1,1	4,1	No
patient-255	nd	-	-	-	A	-	-	-	-	-	-	m	0201	1101	1501	2705	D	3903	5379	neg	pos	neg	pos	neg	neg	158	56	0,73	0,9	4,3	Yes
patient-256	nd	-	-	-	-	-	-	-	-	-	-	m	0201	2402	1501	4402	D	1342	3232	neg	nd	neg	pos	neg	neg	22	21	0,51	1,1	4,7	No
patient-257	nd	-	-	-	-	-	-	-	-	-	-	m	0204	0301	1501	5101	D	19399	1313	neg	nd	neg	pos	neg	neg	25	28	0,5	1	4,1	No
patient-258	nd	-	-	-	V	-	-	-	-	-	-	m	0201		1501	4002	D	162	400	neg	nd	neg	pos	neg	neg	29	43	0,26	1	4,7	No
patient-259	nd	-	-	-	-	-	-	-	-	-	-	m	0301	2402	1501	3501	D	188	46546	pos	nd	neg	pos	neg	neg	46	43	0,52	1,1	4,3	Yes
patient-260	nd	-	-	-	-	-	-	-	-	-	-	w	0201	2402	1501	1801	D	654250	nd	neg	nd	neg	pos	neg	neg	39	59	0,5	1,13	4,2	No
patient-261	nd	-	-	-	-	-	-	-	-	-	-	m	0301	2402	1501	2705	D	14766	nd	neg	nd	neg	pos	neg	neg	22	28	0,5	1,06	2,5	No
patient-262	nd	-	-	-	-	-	-	-	-	-	-	w	0201	0301	1501	4002	D	300000000	14826	neg	nd	neg	pos	neg	neg	111	221	nd	nd	nd	Yes
patient-263	nd	-	-	-	-	-	-	-	-	-	-	w	0201	6601	1501	5101	D	1023	nd	neg	nd	neg	pos	neg	neg	nd	20	2	nd	nd	No
patient-264	nd	-	-	-	A/S	-	-	-	-	-	I	m	0201	2901/02	1503	4402	D	7000000	15459	neg	pos	neg	pos	neg	neg	511	377	1,39	1,3	2,7	Yes
patient-265	nd	-	-	-	T	-	-	-	-	-	-	m	1101	3101	1508	3501	D	1792	18547	neg	pos	neg	pos	neg	neg	26	34	0,55	1	4,9	No
patient-266	nd	-	-	-	-	-	-	-	-	-	-	w	2301	3301	1510	4403	A	6642	nd	neg	nd	neg	pos	neg	neg	nd	21	17	nd	nd	No
patient-267	nd	-	-	-	-	-	-	-	-	-	Y	m	0301		1517	5101	D	276317	9727	neg	nd	neg	pos	neg	neg	40	60	0,31	1,1	4,7	Yes
patient-268	nd	-	-	-	H	-	-	Y	-	-	-	w	0201		1517	5701	D	930	nd	neg	nd	neg	pos	neg	neg	nd	20	nd	1,02	nd	No
patient-269	nd	-	-	-	-	-	-	-	-	-	-	m	3201	6801	1518	3801	D	7000000	124925	pos	nd	neg	pos	neg	neg	98	196	0,41	1	4	Yes
patient-270	nd	-	-	-	-	-	-	-	-	-	-	m	0101	0302	1518	5101	D	4294893	nd	pos	nd	neg	pos	neg	neg	36	67	nd	nd	nd	Yes
patient-271	nd	-	-	-	T	-	-	-	-	-	-	m	2601	3402	1801	7801	A	1061247	1002	neg	pos	neg	pos	neg	neg	200	375	0,48	1,3	4,7	Yes
patient-272	nd	-	-	-	H	-	-	Y	-	-	-	w	0201	2301	1801	4403	A	1464	12368	neg	nd	neg	pos	neg	neg	25	27	0,65	1	4,5	No
patient-273	nd	-	-	-	-	-	-	-	-	-	-	m	205/08	3002	1801	5001	A	88800	8147	pos	nd	neg	pos	neg	neg	105	74	nd	nd	nd	No
patient-274	nd	-	-	-	-	-	-	-	-	-	-	w	0101	0301	1801	4006	D	7000000	124925	pos	neg	neg	pos	neg	neg	28	28	0,21	1	3,6	Yes
patient-275	nd	-	-	-	-	-	-	-	-	-	-	m	0201	2301	1801	4403	D	7000000	2	pos	nd	neg	pos	neg	neg	31	21	0,92	1,1	4,8	Yes
patient-276	nd	-	-	-	T	-	-	-	-	-	-	m	0201		1801	5101	D	73	8937	neg	nd	neg	pos	neg	neg	nd	nd	nd	nd	nd	No
patient-277	nd	-	-	-	-	-	-	-	-	-	I	m	0201	0301	1801	4901	D	1999994,8	5615,42	neg	nd	neg	pos	neg	neg	61	98	0,5	1,06	nd	No
patient-278	nd	-	-	-	-	-	-	-	-	-	-	w	2402	3201	1801	4002	D	3796	nd	neg	nd	neg	pos	neg	neg	25	34	0,6	nd	nd	No
patient-279	nd	-	-	-	-	-	-	-	-	-	-	m	1101	2402	1801	3701	D	740000	nd	neg	nd	neg	pos	neg	neg	33	114	0,6	1	4,6	No
patient-280	nd	-	-	-	-	-	-	-	-	-	-	m	3002	6801	1801	4001	D	8912009	nd	neg	nd	neg	pos	neg	neg	86	146	nd	1,09	7,9	No
patient-281	nd	-	-	-	-	-	-	-	-	-	I	m	0201	0301	1801	4901	D	86995	nd	neg	nd	neg	pos	neg	neg	47	47	1	1,01	nd	No
patient-282	nd	-	-	-	-	-	-	-	-	-	-	m	2501	3201	1801	5701	D	3101	nd	neg	nd	neg	pos	neg	neg	29	46	0,7	1,03	4,9	No
patient-283	nd	-	-	-	A	-	-	-	-	-	-	w	0101	1101	1801	3502	D	21727	nd	neg	nd	neg	pos	neg	neg	37	41	0,9	1,16	4,7	No
patient-284	nd	-	-	-	-	-	-	-	-	-	-	w	0301	1101	1801	4006	D	11979	nd	neg	nd	neg	pos	neg	neg	39	55	0,5	1,05	nd	No
patient-285	nd	-	-	-	-	-	-	Y	-	N	-	w	0201		1801	4402	D	2425	nd	neg	nd	neg	pos	neg	neg	31	nd	0,2	0,99	4,6	No
patient-286	nd	-	-	-	-	-	-	-	-	-	-	w	0301	2402	1801	5201	D	150000	nd	neg	nd	neg	pos	neg	neg	37	34	0,3	0,99	4,6	No
patient-287	nd	-	-	-	-	-	-	-	-	-	-	m	1101	6801	1801	4001	D	393	nd	pos	nd	neg	pos	neg	neg	44	64	0,5	0,99	4,3	No
patient-288	nd	-	-	-	-	-	-	-	-	-	-	m	3002	6801	1801	2705	D	214	nd	neg	nd	neg	pos	neg	neg	24	30	0,4	1,03	nd	No
patient-289	nd	-	-	-	-	-	-	-	-	-	-	m	1101	2301	1801	4403	D	36067520	38965,59	pos	nd	neg	pos	neg	neg	67	93	nd	nd	nd	Yes
patient-290	nd	-	-	-	-	-	-	-	-	-	-	nd	2901/02	6801	1801	4403	D	6713	965	neg	nd	neg	pos	neg	neg	26	76	nd	nd	nd	No
patient-291	nd	-	-	-	A/S	-	-	-	-	-	-	m	1101	2402	1801	3501	D	2000	nd	neg	nd	neg	pos	neg	neg	37	66	nd	nd	nd	No
patient-292	nd	-	-	-	-	-	-	-	-	-	-	w	0301	1101	1805	3501	D	134,8	846,01	neg	nd	neg	pos	neg	neg	33	82	nd	0,99	nd	No

patient-293	nd	-	-	-	T	-	-	-	-	-	-	m	0201	6601	2702	5102	D	135519	3088,08	neg	nd	neg	pos	neg	neg	nd	151	0,3	1,09	3,8	No
patient-294	nd	-	-	-	-	-	-	-	-	-	-	m	0301	0301	2702	4101	D	1361	nd	neg	nd	neg	pos	neg	neg	38	70	0,6	1,03	nd	Yes
patient-295	nd	-	-	-	-	-	-	-	-	-	-	m	0301	6802	2702	3801	D	54475	nd	neg	nd	neg	pos	neg	neg	41	96	0,4	1,06	4,6	Yes
patient-296	nd	-	-	-	-	-	-	-	-	-	-	w	2402	2601	2702	2705	D	304380	nd	pos	nd	neg	pos	neg	neg	45	99	0,5	0,95	nd	Yes
patient-297	nd	-	-	-	-	-	-	-	-	-	-	w	0301	6801	2702	5701	D	44880	nd	neg	nd	neg	pos	neg	neg	24	36	0,5	1	4,6	No
patient-298	nd	-	-	-	-	-	-	-	-	-	-	w	2402	3101	2705	5001	A	1613	15772	neg	nd	neg	pos	neg	neg	14	6	0,23	1	4,2	No
patient-299	nd	-	-	-	H	-	-	-	-	-	-	m	2402	2902	2705	4403	A	1138,6	7316,44	neg	nd	neg	pos	neg	neg	43	73	0,4	0,97	nd	No
patient-300	nd	-	-	-	E	-	-	-	-	-	-	m	2402	6802	2705	4405	D	15310	34	neg	nd	neg	pos	neg	neg	96	209	0,73	1,1	4,3	No
patient-301	nd	-	-	-	-	-	-	-	-	-	-	m	0201		2705	3801	D	47384	nd	neg	nd	neg	pos	neg	neg	41	96	0,7	1,05	nd	Yes
patient-302	nd	-	-	-	-	-	-	-	-	-	-	w	2402		2707	3502	D	1227	3218	neg	pos	neg	pos	neg	neg	18	19	0,44	1,1	4,6	No
patient-303	nd	-	-	-	-	-	-	-	-	-	-	m	2402	2902	3501	4403	A	300,2	6856,4	neg	nd	neg	pos	neg	neg	48	43	0,4	1	7,8	No
patient-304	nd	-	-	-	A	-	-	-	-	-	-	m	0205	1101	3501	5001	D	839	233	neg	nd	neg	pos	neg	neg	23	27	0,54	1	nd	No
patient-305	nd	-	-	-	-	-	-	-	-	-	-	w	0301	6601	3501	4102	D	14115	2002	neg	nd	neg	pos	neg	neg	25	24	0,22	1	nd	No
patient-306	nd	-	-	-	A	-	-	F/Y	-	-	-	w	0201	0301	3501	4402	D	2249	3003	neg	nd	neg	pos	neg	neg	29	21	0,29	1	4,5	No
patient-307	nd	-	-	-	-	-	-	-	-	-	-	m	2402	3001	3501	3503	D	904	nd	neg	nd	neg	pos	neg	neg	40	83	0,95	1	nd	Yes
patient-308	nd	-	-	-	A	-	-	-	-	-	-	m	0205	1101	3501	5001	D	459	187	neg	nd	neg	pos	neg	neg	27	35	0,34	1	4,4	No
patient-309	nd	-	-	-	-	-	-	-	-	-	-	m	1101	6601	3501	4102	D	1257,4	2793,06	neg	nd	neg	pos	neg	neg	32	36	0,6	1,06	nd	No
patient-310	nd	-	-	-	-	-	-	Y	-	-	-	w	0201	1101	3501	4001	D	1285	nd	neg	nd	neg	pos	neg	neg	18	11	0,3	0,98	nd	No
patient-311	nd	-	-	-	-	-	-	-	-	-	-	w	0301	1101	3501	3701	D	8598533	nd	neg	nd	neg	pos	neg	neg	175	513	0,4	1,06	4,3	No
patient-312	nd	-	-	-	-	-	-	-	-	-	-	m	0101	1101	3501	3502	D	1200000	nd	pos	nd	neg	pos	neg	neg	32	82	0,8	1,1	4,6	No
patient-313	nd	-	-	-	T	-	-	Y	-	-	-	m	0201		3501	5201	D	20	nd	pos	nd	neg	pos	neg	neg	32	77	1,1	1,1	nd	No
patient-314	nd	-	-	-	-	-	-	-	-	-	-	w	0301	2402	3501	3501	D	24553	nd	neg	nd	neg	pos	neg	neg	nd	19	nd	1,05	nd	No
patient-315	nd	-	-	-	T	-	-	-	-	-	-	w	0301	3101	3501	5701	D	976	250	neg	nd	neg	pos	neg	neg	nd	23	nd	1,03	nd	No
patient-316	nd	-	-	-	A/S	-	-	-	-	-	-	m	0201	2402	3501	4405	D	8190	nd	neg	nd	neg	pos	neg	neg	61	86	0,3	nd	nd	Yes
patient-317	nd	-	-	-	A	-	-	-	-	-	-	m	0302	1101	3501	4102	D	2399	nd	neg	nd	neg	pos	neg	neg	20	27	0,5	nd	4,7	Yes
patient-318	nd	-	-	-	-	-	-	-	-	-	V/L	m	1101	6801	3501	3503	D	75952	nd	pos	nd	neg	pos	neg	neg	30	58	nd	nd	nd	Yes
patient-319	nd	-	-	-	-	-	-	-	-	-	-	m	2402	3201	3501	4402	D	1113989	nd	neg	nd	neg	pos	neg	neg	80	296	0,5	1,01	4,9	No
patient-320	nd	-	-	-	-	-	-	-	-	-	-	w	0201	3204	3501	4001	D	2833892	nd	pos	nd	neg	pos	neg	neg	31	18	1,1	0,86	4,3	Yes
patient-321	nd	-	-	-	-	-	-	-	-	-	-	w	1101	2601	3501		D	5321	nd	pos	nd	neg	pos	neg	neg	28	23	0,3	1,08	4,6	Yes
patient-322	nd	-	-	-	A/S	-	-	-	-	-	-	w	1101	3303	3501		D	2199	nd	neg	nd	neg	pos	neg	neg	18	19	0,3	1,08	nd	No
patient-323	nd	-	-	-	-	-	-	-	-	-	-	w	3002	3201	3501	4901	D	957	nd	neg	nd	neg	pos	neg	neg	nd	21	nd	nd	nd	No
patient-324	nd	-	-	-	-	-	-	-	-	-	-	w	1101	2901/02	3501	4403	D	155800	129	pos	nd	neg	pos	neg	neg	24	33	nd	nd	nd	Yes
patient-325	nd	-	-	-	-	-	-	-	-	-	-	w	1101	3101	3501	3701	D	340000	1880	neg	nd	neg	pos	neg	neg	30	47	nd	nd	nd	No
patient-326	nd	-	-	-	-	-	-	-	-	-	-	w	0201	1101	3501	4402	D	8000000	8428	neg	nd	neg	pos	neg	neg	73	80	nd	nd	nd	No
patient-327	nd	-	-	-	-	-	-	-	-	-	-	w	1101	3201	3501	5501	D	1295	nd	neg	nd	neg	pos	neg	neg	nd	22	16	nd	nd	No
patient-328	nd	-	-	-	-	-	-	-	-	-	-	m	0201	6901	3502	4901	D	154465	11704	neg	nd	neg	pos	neg	neg	32	63	0,68	1	4,6	No
patient-329	nd	-	-	-	-	-	-	-	-	-	-	w	2402		3502		D	3774	nd	neg	nd	neg	pos	neg	neg	36	32	0,3	0,94	nd	Yes
patient-330	nd	-	-	-	P	-	-	-	-	-	-	m	2901	3201	3502	3508	D	216093	nd	neg	nd	neg	pos	neg	neg	39	50	0,5	1,06	nd	No
patient-331	nd	-	-	-	-	-	-	-	-	-	-	m	0301	6801	3503	4402	A	2219	4378	neg	pos	neg	pos	neg	neg	36	65	0,74	1,2	3,9	No
patient-332	nd	-	-	-	T	-	-	-	-	-	I	m	1101	6801	3503	5601	A	1886	32995	neg	pos	neg	pos	neg	neg	30	42	0,47	1	4,9	No
patient-333	nd	-	-	-	P	-	-	-	-	-	-	w	1101	3301/03	3503	5601	A	2407	nd	neg	nd	neg	pos	neg	neg	nd	24	5	nd	nd	No
patient-334	nd	-	-	-	-	-	-	-	-	-	-	m	2402	6801	3503	5107	D	2090	718	neg	nd	neg	pos	neg	neg	28	36	0,39	0,9	4,3	No
patient-335	nd	-	-	-	P	-	-	-	-	-	-	m	2402		3503		D	7000000	10209	pos	neg	neg	pos	neg	neg	191	474	0,87	1,1	3,8	Yes
patient-336	nd	-	-	-	A	-	-	-	-	-	-	w	0201	2405	3503	4002	D	1587	666	neg	pos	neg	pos	neg	neg	42	34	0,59	1,2	4	No
patient-337	nd	-	-	-	-	-	-	-	-	-	-	w	2402	2601	3503	4102	D	4770	5519	neg	nd	neg	pos	neg	neg	29	34	73	1	4,3	No
patient-338	nd	-	-	-	-	-	-	-	-	A	-	m	0201	0301	3503	5101	D	61398686	nd	neg	nd	neg	pos	neg	neg	80	226	0,6	1,03	4,6	Yes
patient-339	nd	-	-	-	A	-	-	-	-	-	-	w	0101	0201	3503	3801	D	534	nd	neg	nd	neg	pos	neg	neg	20	18	0,4	0,99	4,5	No
patient-340	nd	-	-	-	-	-	-	-	-	-	-	m	1101	3101	3503	5201	D	446745506	nd	pos	nd	neg	pos	neg	neg	230	725	1,1	nd	4,7	No
patient-341	nd	-	-	-	T	-	-	-	-	N	-	m	0101	0201	3503	5101	D	35590	nd	neg	nd	neg	pos	neg	neg	71	207	nd	0,95	nd	Yes
patient-342	nd	-	-	-	-	-	-	-	-	-	-	w	2402	3201	3503	3508	D	8299	nd	neg	nd	neg	pos	neg	neg	28	20	2,5	1,06	4,3	No
patient-343	nd	-	-	-	T	-	-	-	-	-	-	m	0101		3503	4006	D	413000	nd	neg	nd	neg	pos	neg	neg	70	80	nd	nd	nd	Yes
patient-344	nd	-	-	-	A	-	-	-	-	-	-	m	2402		3503	3506	D	5217	807	neg	nd	neg	pos	neg	neg	nd	38	8	nd	nd	No
patient-345	nd	-	-	-	T	-	-	-	-	-	-	m	0201	1101	3503	5101	D	513	nd	neg	nd	neg	pos	neg	neg	nd	31	10	nd	nd	No
patient-346	nd	-	-	-	A	-	-	-	-	-	-	w	0301	3101	3506	4001	A	977	30859	neg	nd	neg	pos	neg	neg	nd	21	8	nd	nd	No
patient-347	nd	-	-	-	-	-	-	-	-	-	-	m	2301	2402	3508	5001	A	2562	982	neg	nd	neg	pos	neg	neg	30	25	0,5	1,87	4,5	No
patient-348	nd	-	-	-	-	-	-	-	-	-	-	w	0101	3004	3508	5101	D	4081	nd	pos	nd	neg	pos	neg	neg	18	15	nd	1,04	nd	No
patient-349	nd	-	-	-	V	-	-	-	-	-	I	w	0201	0301	3701	5201	D	3187	1325	neg	pos	neg	pos	neg	neg	17	16	0,63	1	4,7	No
patient-350	nd	-	-	-	-	-	-	-	-	-	-	m	0101	0301	3701	5101	D	4853	nd	neg	nd	neg	pos	neg	neg	36	53	1,2	0,99	5	No
patient-351	nd	-	-	-	-	-	-	-	-	-	-	m	206/10	1101	3701	5701	D	6166	nd	neg	nd	neg	pos	neg	neg	nd	23	7	nd	nd	No
patient-352	nd	-	-	-	-	-	-	-	-	-	-	w	0101	0301	3801	4403	A	4365	nd	neg	nd	neg	pos	neg	neg	nd	27	3	nd	nd	No
patient-353	nd	-	-	-	A	-	-	-	-	-	-	w	0101	3004	3801	4501	D	5585	957	neg	nd	neg	pos	neg	neg	20	16	0,26	0,9	4,3	No
patient-354	nd	-	-	-	-	-	-	-	-	-	-	m	2301	2601	3801	4403	D	1007827	2849	pos	nd	neg	pos	neg	neg	37	49	0,51	1	4,6	Yes
patient-355	nd	-	-	-	-	-	-	Y	-	-	-	w	0201	3001	3801	5601	D	3712	301	neg	pos	neg	pos	neg	neg	25	30	0,42	1	4,1	Yes
patient-356	nd	-	-	-	A/V	-	-	-	-	P	-	m	0201	2601	3801	5101	D	9742	nd	neg	nd	neg	pos	neg	neg	nd	83	0,5	1,04	4,5	No
patient-357	nd	-	-	-	V	-	-	-	-	-	-	m	0201	2601	3801	4402	D	1183	nd	neg	nd	neg	pos	neg	neg	nd	68	18	nd	nd	No
patient-358	nd	-	-	-	-	-	-	-	-	-	-	w	3301/03	3401	3802	4002	A	18800	573	neg	nd	neg	pos	neg	neg	51	83	nd	nd	nd	No
patient-359	nd	-	-	-	-	-	-	-	-	-	-	m	0201	2301	3901	4901	D	1829	6669	neg	pos	neg	pos	neg	neg	18	18	1,01	1	4,6	No
patient-360	nd	-	-	-	A	-	-	-	-	-	A	m	0201	2402	3901	5101	D	498	6049,77	neg	nd	neg	pos	neg	neg	39	70	0,8	1,1	4,8	No
patient-361	nd	-	-	-	-	-	-	-	-	A	-	m	0201	3201	3901	5101	D	20	nd	neg	nd	neg	pos	neg	neg	nd	nd	nd	0,93	nd	No
patient-362	nd	-	-	-	-	-	-	Y	-	-	-	m	0101	0201	3901	5701	D	6526	nd	neg	nd	neg	pos	neg	neg	14	14	0,3	1,03	nd	No
patient-363	nd	-	-	-	-	-	-	-	-	-	-	w	0201	1101	3901	5501	D	2138	5610	neg	nd	neg	pos	neg	neg	nd	46	7	nd	nd	No
patient-364	nd	-	-	-	-	-	-	-	-	-	-	m	0201		3906	5101	A	474	20365	neg	nd	neg	pos	neg	neg	44	40	0,35	2,5	4,3	No
patient-365	nd	-	-	-	A	-	-	-	-	-	I	m	1101	2402	3906	4001	A	1257	nd	neg	nd	neg	pos	neg	neg	nd	26	8	nd	nd	No
patient-366	nd	-	-	-	L	-	-	-	-	-	A	m	0201	3004	4001	5101	A	11749	nd	neg	nd	neg	pos	neg	neg	nd	48	9	nd	nd	No
patient-367	nd	-	-	-	-	-	-	-	-	-	-	w	0201	6801	4001	4901	D	1938	638	neg	pos	neg	pos	neg	neg	18	18	0,28	1,1	4,8	No
patient-368	nd	-	-	-	H	-	-	Y	-	-	-	m	0201	3201	4002	5101	A	141	1255	neg	nd	neg	pos	neg	neg	19	23	1,25	1	4,8	Yes
patient-369	nd	-	-	-	A	-	-	-	-	-	-	w	0101	0201	4002	5701	D	761,2	6828,51	neg	nd	neg	pos	neg	neg	31	35	0,3	0,9	4,5	No
patient-370	nd	-	-	-	-	-	-	-	-	-	-	m	0201	3201	4002	5101	D	831463225	nd	pos	nd	neg	pos	neg	neg	146	172	0,6	1,14	nd	No
patient-371	nd	-	-	-	-	-	-	-	-	-	-	m	2301	6801	4006	4901	D	7654	10060	neg	nd	neg	pos	neg	neg	25	43	0,62	1	4,5	Yes
patient-372	nd	-	-	-	-	-	-	-	-	-	-	m	0101	0201	4006		D	1782	nd	pos	nd	neg	pos	neg	neg	43	66	0,6	nd	4,7	Yes
patient-373	nd	-	-	-	V	-	-	-	-	-	-	w	0101	0201	4006	4101	D	828919	nd	neg	nd	neg	pos	neg	neg	61	87	0,8	nd	4,4	No
patient-374	nd	-	-	-	-	-	-	-	-	-	-	w	0101		4006		D	9812	nd	pos	nd	neg	pos	neg	neg	30	39	0,3	1,05	4,5	Yes
patient-375	nd	-	-	-	-	-	-	-	-	-	-	m	0211	3301/03	4006	4403	D	242	nd	neg	nd	neg	pos	neg	neg	nd	24	9	nd	nd	No
patient-376	nd	-	-	-	-	-	-	-	-	-	-	m	2402	3301/03	4006	5801	D	2673	nd	neg	nd	neg	pos	neg	neg	nd	17	9	nd	nd	No
patient-377	nd	-	-	-	A	-	-	-	-	-	-	m	0202	0301	4101	5801	A	1326	11029	neg	pos	neg	pos	neg	neg	25	44	0,15	0,9	4,5	No
patient-378	nd	-	-	-	A	-	-	-	-	-	I	m	0101	0201	4101	5001	D	1613	10156	neg	pos	neg	pos	neg	neg	34	52	0,53	0,9	4,8	No
patient-379	nd	-	-	-	E	-	-	-	-	-	-	m	0101		4101	5101	D	6711326	9950	neg	pos	neg	pos	neg	neg	107	171	0,35	1	4,6	Yes
patient-380	nd	-	-	-	A	-	-	-	-	-	-	m	0201	2301	4101	5001	D	275	4648	neg	nd	neg	pos	neg	neg	24	29	0,45	1	nd	No
patient-381	nd	-	-	-	A/S	-	-	-	-	-	-	m	0202	2301	4101	5703	D	6843	nd	neg	nd	neg	pos	neg	neg	28	41	0,8	1,08	4,5	No
patient-382	nd	-	-	-	-	-	-	-	-	-	-	w	3001		4101		D	62138	nd	neg	nd	neg	pos	neg	neg	60	57	nd	nd	nd	Yes
patient-383	nd	-	-	-	-	-	-	-	-	-	-	m	0101		4101	5801	D	4690	nd	neg	nd	neg	pos	neg	neg	64	98	0,5	1,06	4,6	No
patient-384	nd	-	-	-	V	-	-	-	-	-	-	w	2402	6601	4102	5101	D	29625	nd	neg	nd	neg	pos	neg	neg	29	44	0,5	1	nd	No
patient-385	nd	-	-	-	-	-	-	-	-	-	-	m	0101	6601	4102	5701	D	1339	40786,84	neg	nd	neg	pos	neg	neg	55	56	0,6	nd	nd	No
patient-386	nd	-	-	-	T	-	-	-	-	-	-	w	0301	2402	4102	5101	D	631	nd	neg	nd	neg	pos	neg	neg	nd	65	17	nd	nd	No
patient-387	nd	-	-	-	A	-	-	F/Y	-	-	-	w	0101	3001	4201	5301	D	26942	nd	neg	nd	neg	pos	neg	neg	50	59	1,2	1,08	nd	No
patient-388	nd	-	-	-	-	-	-	-	-	-	-	w	0301	3201	4402	4403	A	992	5259	neg	nd	neg	pos	neg	neg	21	21	0,8	0,98	4,8	No
patient-389	nd	-	-	-	-	-	-	Y	-	-	-	w	0201		4402	5301	D	20989	14935	neg	neg	neg	pos	neg	neg	22	17	0,44	1,1	4,2	Yes
patient-390	nd	-	-	-	A	-	-	-	-	-	-	m	0301	0302	4402	5101	D	2071	nd	neg	nd	neg	pos	neg	neg	49	85	0,4	0,95	nd	No
patient-391	nd	-	-	-	V	-	-	-	-	T	-	w	0101	0201	4402	5201	D	628	518	neg	nd	neg	pos	neg	neg	nd	nd	nd	0,98	4,5	No
patient-392	nd	-	-	-	A/S	-	-	-	-	-	-	w	2402	3201	4402	5101	D	3388	nd	neg	nd	neg	pos	neg	neg	20	27	0,6	1,03	6,2	No
patient-393	nd	-	-	-	A	-	-	-	-	-	-	w	2402	2501	4402	5101	D	9772	58050	neg	nd	neg	pos	neg	neg	nd	34	5	nd	nd	No
patient-394	nd	-	-	-	-	-	-	-	-	-	-	m	1101	2301	4403	5501	A	639	nd	neg	nd	neg	pos	neg	neg	21	26	0,65	1,1	4	No
patient-395	nd	-	-	-	H	-	-	Y	-	-	-	m	0201		4403	5701	A	11235,6	6556,6	neg	nd	neg	pos	neg	neg	36	31	nd	nd	nd	No
patient-396	nd	-	-	-	A	-	-	-	-	-	-	w	0101	1101	4403	5701	A	3934	nd	neg	nd	neg	pos	neg	neg	nd	14	nd	nd	nd	No
patient-397	nd	-	-	-	-	-	-	-	-	-	-	m	2402	2901/02	4403	5002	D	6665119	5651	neg	pos	neg	pos	neg	neg	52	123	0,71	1,1	4,5	Yes
patient-398	nd	-	-	-	-	-	-	-	-	-	-	w	0205	0301	4403	5001	D	21616	nd	neg	nd	neg	pos	neg	neg	39	50	0,7	1,03	nd	No
patient-399	nd	-	-	-	A/S	-	-	-	-	-	-	w	0201	0301	4403	4405	D	2092	nd	neg	nd	neg	pos	neg	neg	nd	24	0,3	0,95	nd	No
patient-400	nd	-	-	-	-	-	-	-	-	-	-	w	0201	2301	4403	5101	D	741	nd	neg	nd	neg	pos	neg	neg	56	104	0,5	1,07	nd	Yes
patient-401	nd	-	-	-	-	-	-	-	-	-	-	m	2901/02	3001	4403	5001	D	2809	nd	neg	nd	neg	pos	neg	neg	nd	18	19	nd	nd	No
patient-402	nd	-	-	-	V	-	-	-	-	-	-	m	0201	205/08	4429	5001	D	5030	nd	neg	nd	neg	pos	neg	neg	nd	31	7	nd	nd	No
patient-403	nd	-	-	-	-	-	-	-	-	-	-	w	0205	2402	4901	5001	D	3113600	6597,48	neg	nd	neg	pos	neg	neg	162	294	0,7	1	nd	No
patient-404	nd	-	-	-	G	-	-	-	-	-	-	m	0302	2301	4901	5101	D	210036	nd	neg	nd	neg	pos	neg	neg	nd	43	0,3	nd	nd	No
patient-405	nd	-	-	-	V	-	-	-	-	-	-	m	0201	2301	4901	5201	D	1072	nd	neg	nd	neg	pos	neg	neg	nd	28	27	nd	nd	No
patient-406	nd	-	-	-	T	-	-	-	-	-	-	m	0206	3001	5001	5101	D	7000000	11369	neg	nd	neg	pos	neg	neg	132	203	0,51	1,1	4,1	Yes
patient-407	nd	-	-	-	N	-	-	-	-	-	A	m	1101	6801	5001	5101	D	30900	68	neg	nd	neg	pos	neg	neg	33	38	nd	nd	nd	No
patient-408	nd	-	-	-	G	-	-	-	-	-	-	m	6801		5101		D	38286	9100	neg	pos	neg	pos	neg	neg	28	31	0,3	1	4,1	Yes
patient-409	nd	-	-	-	Q	-	-	-	-	-	-	m	2402	2601	5101	5502	D	283578	2565	neg	pos	neg	pos	neg	neg	54	103	0,94	1	4,4	No
patient-410	nd	-	-	-	-	-	-	-	-	-	-	m	3201	3301/03	5101		D	2230	nd	neg	nd	neg	pos	neg	neg	52	49	0,9	1,2	4,6	Yes
patient-411	nd	-	-	-	Q	-	-	Y	-	-	-	m	2301	2402	5101		D	2770	4039	neg	nd	neg	pos	neg	neg	23	9	1,15	0,9	4,6	No
patient-412	nd	-	-	-	-	-	-	-	-	-	-	w	0201	2402	5101	5201	D	93	nd	neg	nd	neg	pos	neg	neg	17	14	0,31	1	4,2	No
patient-413	nd	-	-	-	-	-	-	-	-	-	-	w	0201	3101	5101	5101	D	10936	nd	neg	nd	neg	pos	neg	neg	27	34	0,4	1	nd	No
patient-414	nd	-	-	-	-	-	-	-	-	-	-	w	1101	2601	5101	5201	D	5728	nd	neg	nd	neg	pos	neg	neg	nd	nd	nd	1,01	nd	No
patient-415	nd	-	-	-	A	-	-	-	-	-	-	w	0201		5101	5801	D	628	nd	neg	nd	neg	pos	neg	neg	20	19	nd	nd	nd	No
patient-416	nd	-	-	-	N	-	-	-	-	-	A	m	1101	2402	5101		D	72000	2023	neg	nd	neg	pos	neg	neg	37	60	nd	nd	nd	No
patient-417	nd	-	-	-	T	-	-	-	-	-	-	m	0101	0301	5101	5701	D	300000000	1398	neg	nd	neg	pos	neg	neg	234	259	nd	nd	nd	Yes
patient-418	nd	-	-	-	-	-	-	-	-	-	-	m	204/17		5101		D	293244800	4848	pos	nd	neg	pos	neg	neg	51	129	nd	nd	nd	No
patient-419	nd	-	-	-	-	-	-	-	-	-	-	m	0201	6801	5101		D	6381	nd	neg	nd	neg	pos	neg	neg	nd	48	10	nd	nd	No
patient-420	nd	-	-	-	-	-	-	-	-	-	I	w	0301	1101	5201	5601	A	2142	nd	neg	nd	neg	pos	neg	neg	nd	36	5	nd	nd	No
patient-421	nd	-	-	-	-	-	-	-	-	-	-	m	0101	0301	5201	5701	D	1549	nd	neg	nd	neg	pos	neg	neg	nd	125	26	nd	nd	No
patient-422	nd	-	-	-	E	-	-	-	-	-	-	w	2901/02	3402	5301		D	1804	1411	neg	nd	neg	pos	neg	neg	15	21	0,31	1	3,5	No
patient-423	nd	-	-	-	Q	-	-	-	-	-	A	m	6802		5301		D	39	402	neg	nd	neg	pos	neg	neg	19	20	0,65	1	4,4	No
patient-424	nd	-	-	-	A/S	-	-	-	-	-	-	w	0201	2402	5701	5801	D	23323	nd	neg	nd	neg	pos	neg	neg	40	54	0,5	1,06	nd	No
patient-425	nd	-	-	-	-	-	-	-	-	-	-	m	0101	3301/03	5701	5801	D	6528000	7304	neg	nd	neg	pos	neg	neg	38	37	nd	nd	nd	No
patient-426	nd	-	-	-	-	-	-	-	-	-	-	m	02xx	03xx	37xx	38xx	A	6859536,8	6703,18	neg	nd	neg	pos	neg	neg	143	98	1,3	1,3	3,3	No
patient-427	nd	-	-	-	-	-	-	-	-	-	-	w	0101		4004/06	4006	D	4417	19652	neg	nd	neg	pos	neg	neg	18	21	nd	1	nd	No
patient-428	nd	-	-	-	-	-	-	-	-	-	-	m	0201	2402	4402/05		A	7000000	825	pos	neg	neg	pos	neg	neg	169	180	5,33	1,3	3,7	Yes
patient-429	nd	-	-	-	-	-	-	-	-	-	-	m	0101	2402	550X	5701	D	2548	nd	neg	nd	neg	pos	neg	neg	23	21	0,4	1,08	0,4	No
patient-430	nd	-	-	-	-	-	-	-	-	-	-	w	2402		xx		D	1407	34826	neg	pos	neg	pos	neg	neg	20	14	0,34	0,9	4,4	No
patient-431	nd	-	-	-	-	-	-	-	-	-	-	m	nd	nd	nd	nd	D	170000000	pos	pos	neg	neg	pos	neg	neg	nd	nd	nd	nd	nd	No
patient-432	nd	-	-	-	-	-	-	-	-	-	-	w	nd	nd	nd	nd	D	170000000	pos	pos	neg	neg	pos	neg	neg	nd	nd	nd	nd	nd	No
patient-433	nd	-	-	-	-	-	-	-	-	-	-	m	nd	nd	nd	nd	D	42961822	pos	pos	neg	neg	pos	neg	neg	nd	nd	nd	nd	nd	No
patient-434	nd	-	-	-	-	-	-	-	-	-	-	m	nd	nd	nd	nd	D	38433580	15516	pos	neg	neg	pos	neg	neg	nd	nd	nd	nd	nd	No
patient-435	nd	-	-	-	-	-	-	-	-	-	-	m	nd	nd	nd	nd	D	30245180	1293	pos	neg	neg	pos	neg	neg	nd	nd	nd	nd	nd	No
patient-436	nd	-	-	-	-	-	-	-	-	-	-	m	nd	nd	nd	nd	D	15545121	pos	pos	neg	neg	pos	neg	neg	nd	nd	nd	nd	nd	No
patient-437	nd	-	-	-	-	-	-	-	-	-	-	w	nd	nd	nd	nd	D	3728603	16246	pos	neg	neg	pos	neg	neg	nd	nd	nd	nd	nd	No
patient-438	nd	-	-	-	-	-	-	-	-	-	-	m	nd	nd	nd	nd	D	1253454	4006	pos	neg	neg	pos	neg	neg	nd	nd	nd	nd	nd	No
patient-439	nd	-	-	-	-	-	-	-	-	-	-	m	nd	nd	nd	nd	D	597821	119	pos	neg	neg	pos	neg	neg	nd	nd	nd	nd	nd	No

patient-440	nd	-	-	-	-	-	-	-	-	-	-	m	nd	nd	nd	nd	D	584640	13204	pos	neg	neg	pos	neg	neg	nd	nd	nd	nd	nd	No
patient-441	nd	-	-	-	-	-	-	-	-	-	-	w	nd	nd	nd	nd	D	388963	pos	pos	neg	neg	pos	neg	neg	nd	nd	nd	nd	nd	No
patient-442	0,86	nd	nd	nd	nd	nd	nd	nd	nd	nd	nd	w	0201	3201	3501	4402	nd	<10	pos	neg	pos	neg	pos	neg	neg	nd	17	0,4	1,01	nd	Yes
patient-443	0,02	nd	nd	nd	nd	nd	nd	nd	nd	nd	nd	w	0101	0101	1402	3501	nd	<11	pos	nd	nd	neg	pos	neg	neg	23	14	0,3	0,98	4,8	Yes
patient-444	1,26	-	-	-	T	-	-	Y	-	-	-	m	0101	0201	0801	3503	D	<10	1369,1	neg	pos	neg	pos	neg	neg	33,28	26	1,1	1,04	4,5	Yes
patient-445	1,98	-	-	-	-	-	-	-	-	-	-	w	3002	3201	3501	4901	D	273	nd	neg	pos	neg	pos	neg	neg	18	16	1	1,09	4,9	No
patient-446	0,037	-	-	-	-	-	-	-	-	-	-	m	0301	2402	1402	3502	D	<10	2884,2	nd	nd	neg	pos	nd	nd	32	46	0,6	1,13	5,3	Yes
patient-447	0,32	-	-	-	-	-	-	-	-	-	-	w	0101	0201	3503	5105	D	543	nd	nd	nd	neg	pos	nd	neg	22	17	0,9	0,98	4,4	No
patient-448	0,22	nd	nd	nd	nd	nd	nd	nd	nd	nd	nd	m	2902	6801	3501	5501	nd	87	nd	neg	pos	neg	pos	neg	neg	32	47	0,4	1,02	9,1	No
patient-449	0,1	nd	nd	nd	nd	nd	nd	nd	nd	nd	nd	w	2402	6801	0705	3503	D	27	pos	neg	pos	neg	pos	neg	neg	32	74	0,4	1,02	4,6	No
patient-450	0,036	-	-	-	-	-	-	-	-	-	-	m	1101	2301	1302	3501	D	14	pos	neg	pos	neg	pos	neg	neg	22	29	0,4	1,7	4,7	Yes
patient-451	0,02	-	-	-	-	-	-	-	-	-	-	w	1101	3303	3501	3501	D	929	pos	neg	pos	neg	pos	neg	neg	20	13	0,3	1,03	4,2	No
patient-452	0,046	-	-	-	-	-	-	-	-	-	-	m	0201	2301	3501	5101	D	<10	7004,37	neg	pos	neg	pos	neg	neg	42	52	0,4	1,06	nd	Yes
patient-453	2,02	nd	nd	nd	nd	nd	nd	nd	nd	nd	nd	m	3601	7401	3501	3901	nd	152	pos	neg	pos	neg	pos	nd	neg	23	31	nd	nd	nd	No
patient-454	0,069	-	-	-	-	-	-	-	-	-	-	f	0301	2402	3501	3501	D	<10	pos	neg	pos	neg	pos	nd	neg	24	17	0,4	1,07	4,7	Yes
patient-455	0,1	-	-	-	-	-	-	-	-	-	-	m	0201	3501	5001	5001	nd	892	nd	nd	nd	neg	pos	neg	nd	24	36	0,5	0,99	4,8	No
patient-456	2,08	nd	nd	nd	nd	nd	nd	nd	nd	nd	nd	m	0201	0301	3503	5001	nd	17	pos	nd	nd	neg	pos	neg		31	32	0,7	0,99	4,4	Yes
patient-457	3,93	-	-	-	-	-	-	-	-	-	-	w	0201	3201	3501	3801	nd	35	nd	neg	pos	neg	pos	neg	neg	18	15	0,4	1,01	4,8	No
patient-458	0,11	nd	nd	nd	nd	nd	nd	nd	nd	nd	nd	w	0201	0302	0801	3501	nd	<10	617,45	nd	nd	neg	pos	neg	nd	nd	nd	nd	nd	nd	Yes
patient-459	0,035	-	-	-	T	-	-	-	-	-	-	w	1101	3101	3501	3502	A	<10	pos	neg	pos	neg	pos	neg	neg	nd	nd	nd	nd	nd	Yes
patient-460	2,68	-	-	-	T	-	-	-	-	-	-	m	0101	0201	3503	5101	D	<10	pos	nd	nd	neg	pos	neg	neg	29	43	0,3	0,98	4,8	Yes
patient-461	0,17	nd	nd	nd	nd	nd	nd	nd	nd	nd	nd	w	0217	2402	3501	4801	nd	12	pos	neg	pos	neg	pos	neg	neg	21	23	0,4	0,99	4,6	Yes
patient-462	nd	-	-	-	-	-	-	-	-	-	-	w	1101	2601	3501	3501	nd	<10	pos	nd	nd	neg	pos	nd	neg	26	20	0,3	1,08	4,8	Yes
patient-463	nd	-	-	-	-	-	-	-	-	-	-	m	0101	0301	1302	3502	D	<10	pos	nd	nd	neg	pos	nd	neg	25	24	0,6	1,06	4,4	Yes
patient-464	nd	-	-	-	-	-	-	-	-	-	-	w	0201	2402	3501	4001	nd	<10	neg	nd	nd	neg	pos	neg	neg	15	13	0,5	nd	4,6	nd
