# Supplementary figures and images for: Immune escape pathways from the HBV core18-27 CD8 T cell response are driven by individual HLA class I alleles
Source: Front Immunol. 2022 Nov 10;13:1045498. doi: 10.3389/fimmu.2022.1045498 (PMC9686862; doi:10.3389/fimmu.2022.1045498)

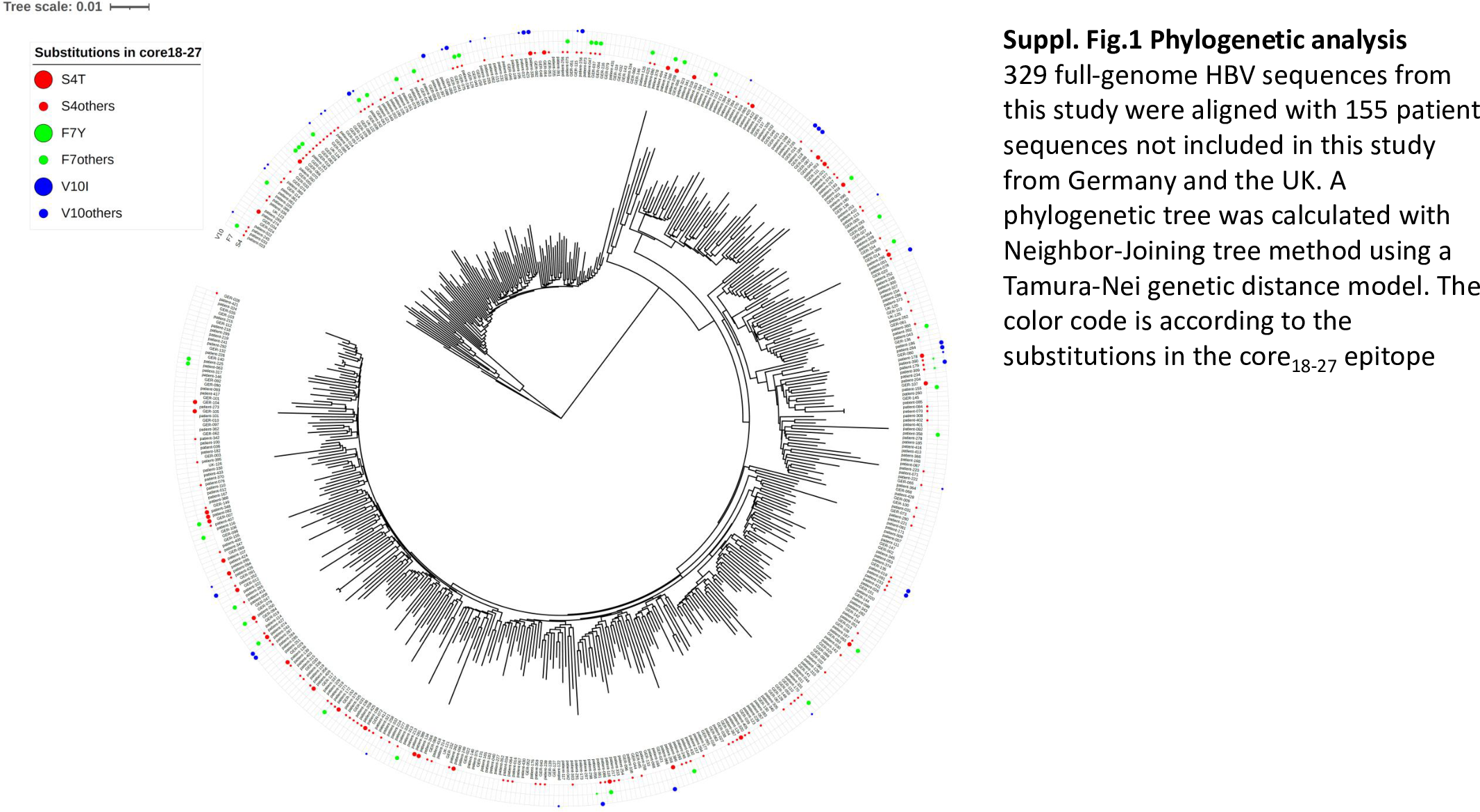

Supplement: Supplementary file 2 [file Image_1.tif]

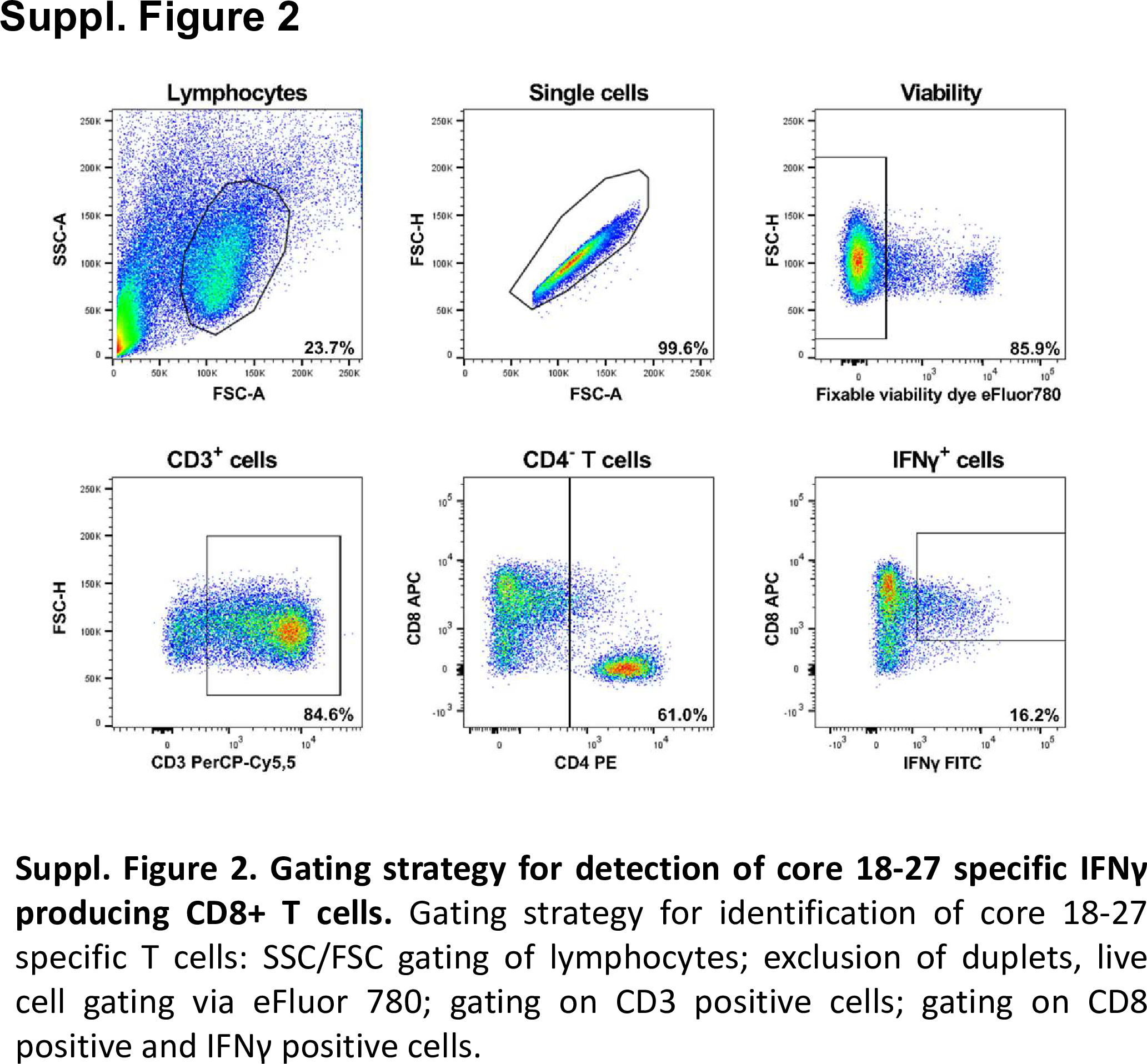

Supplement: Supplementary file 3 [file Image_2.tif]

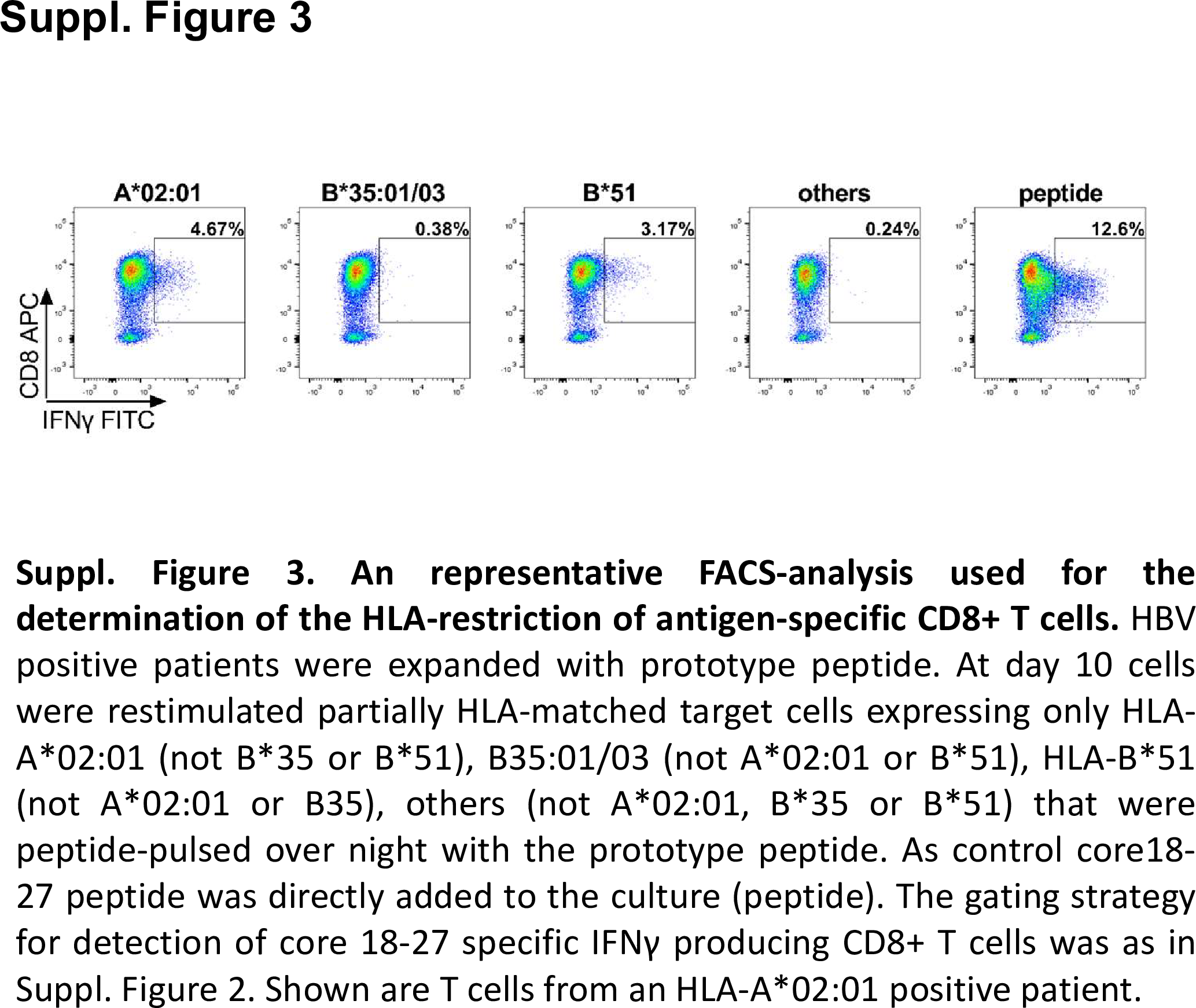

Supplement: Supplementary file 4 [file Image_3.tif]

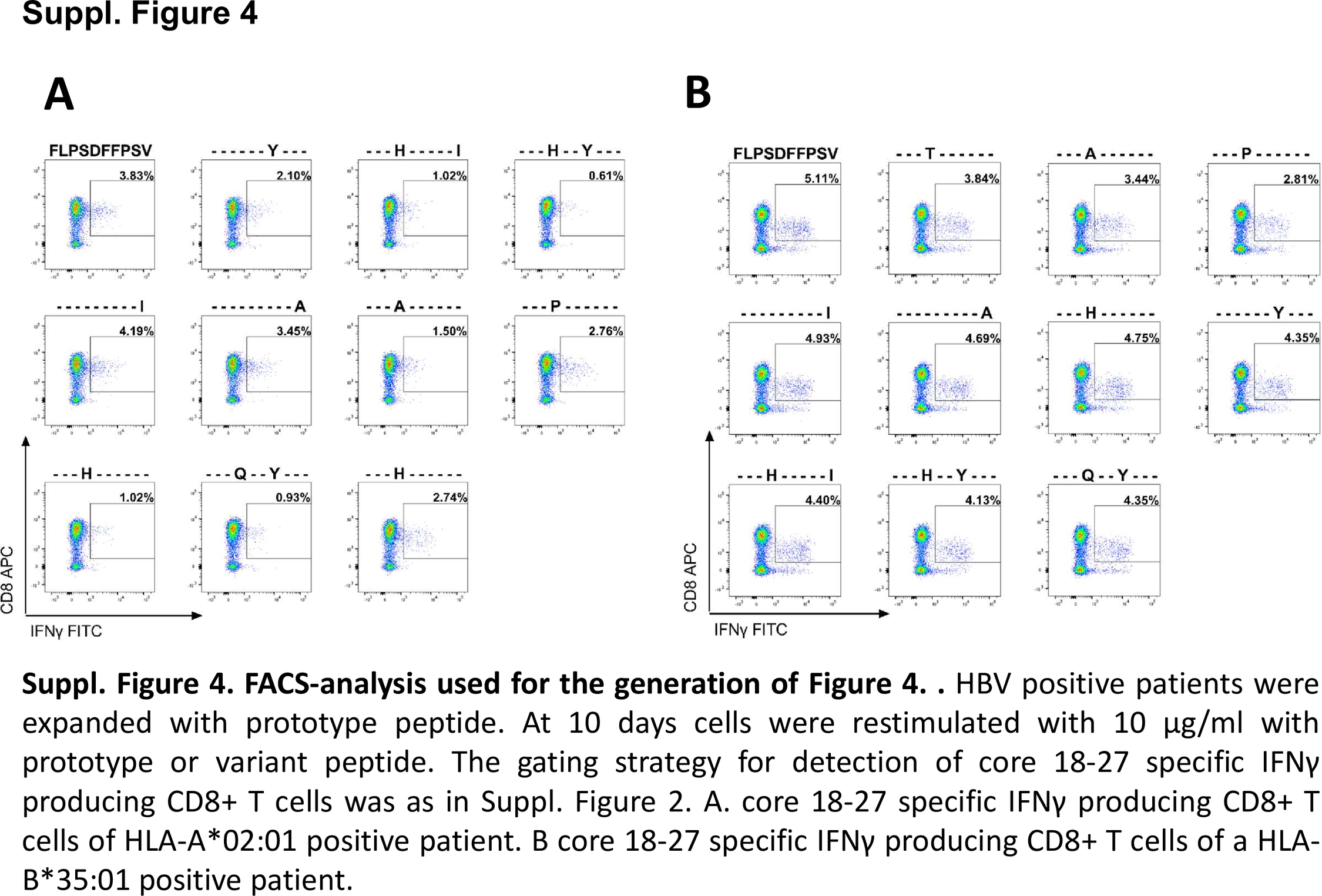

Supplement: Supplementary file 5 [file Image_4.tif]

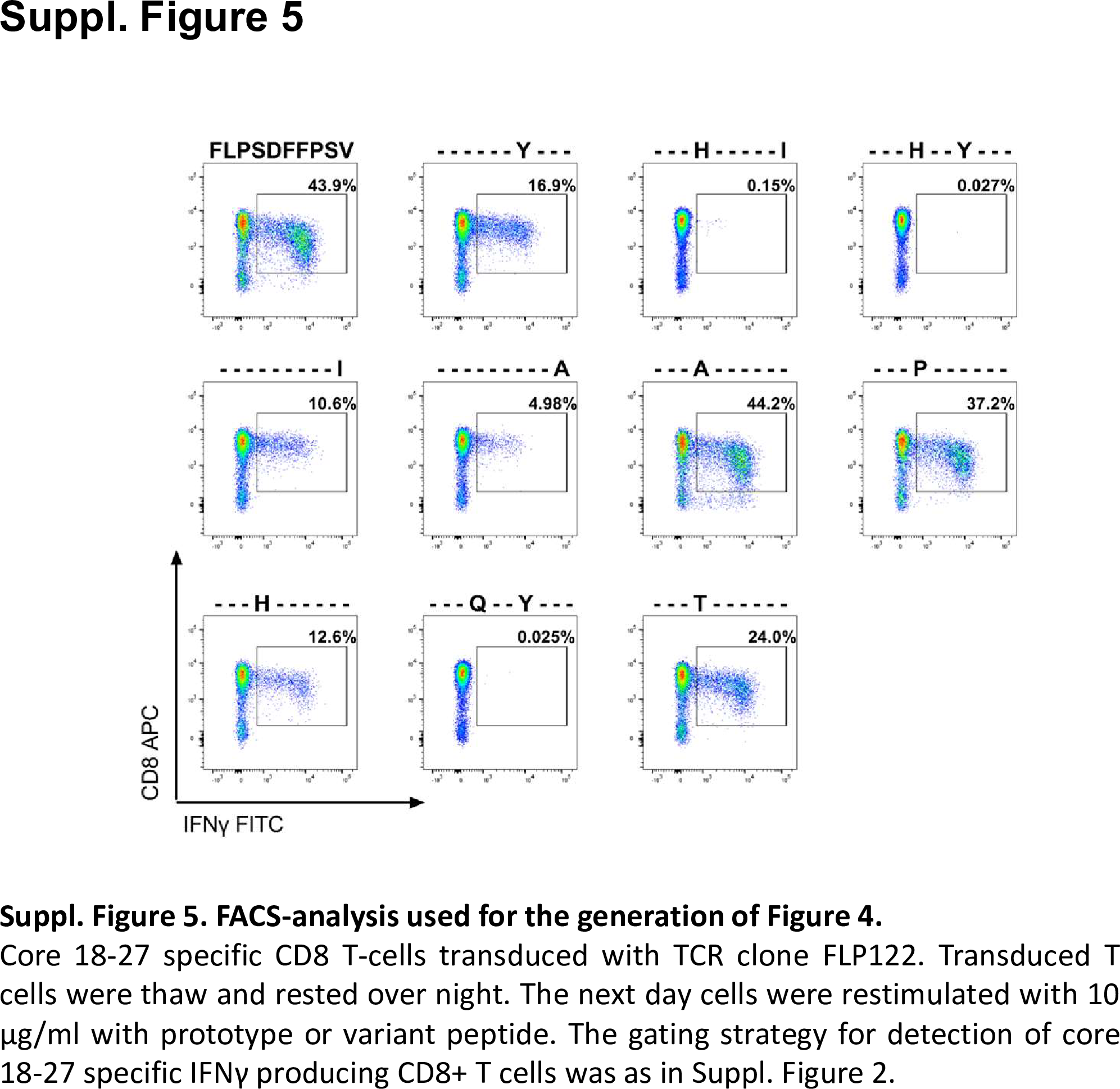

Supplement: Supplementary file 6 [file Image_5.tif]

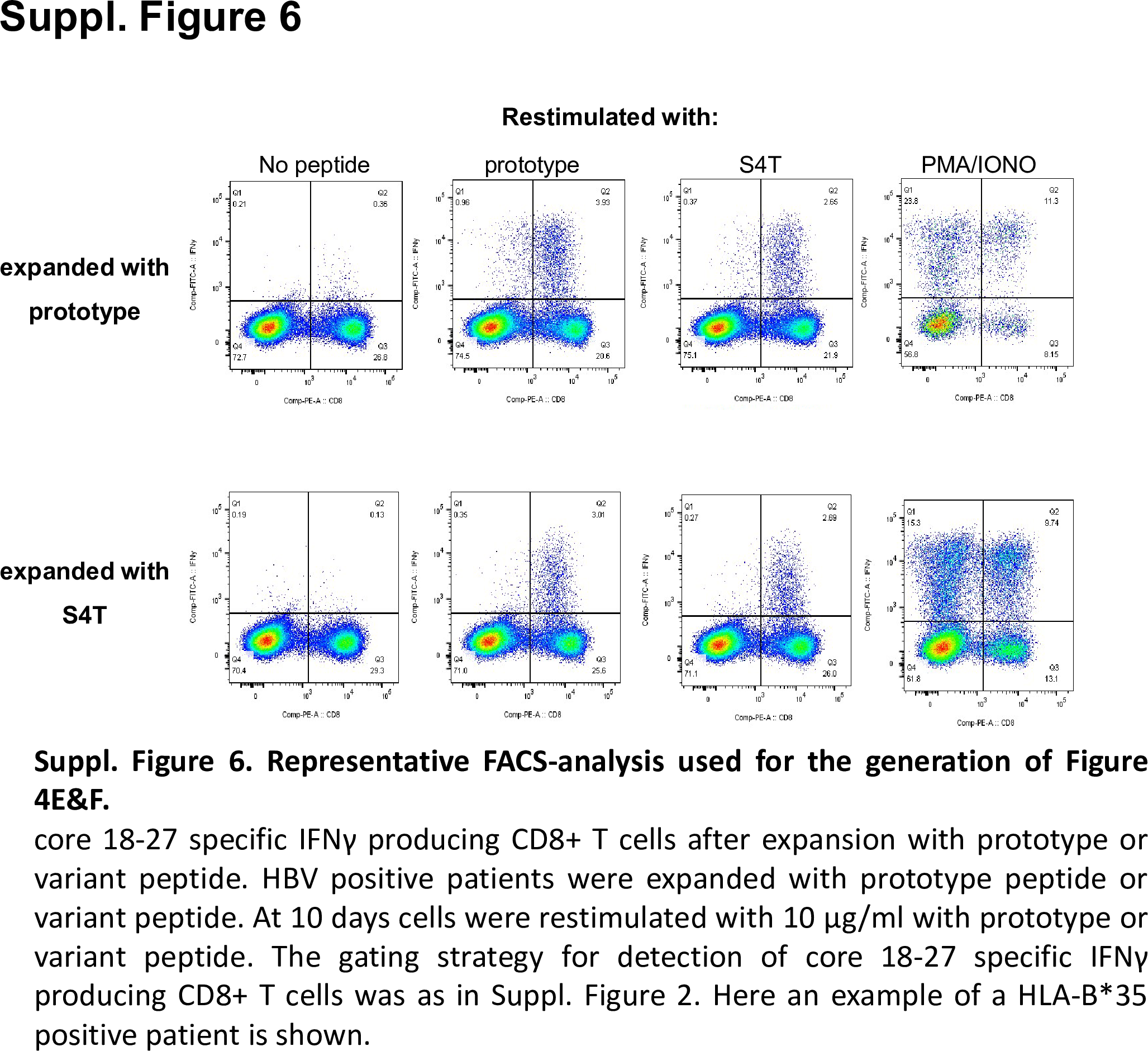

Supplement: Supplementary file 7 [file Image_6.tif]

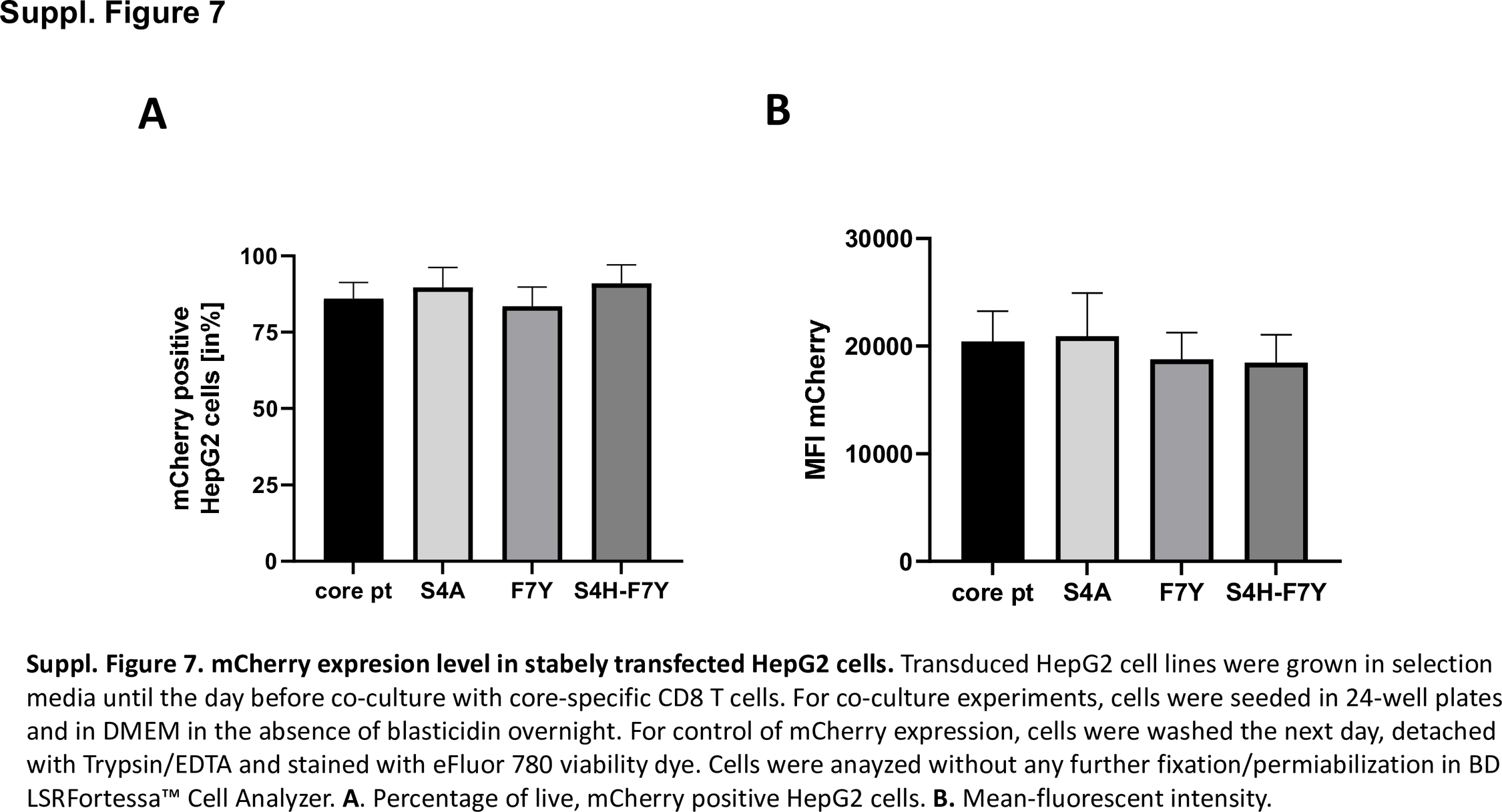

Supplement: Supplementary file 8 [file Image_7.tif]

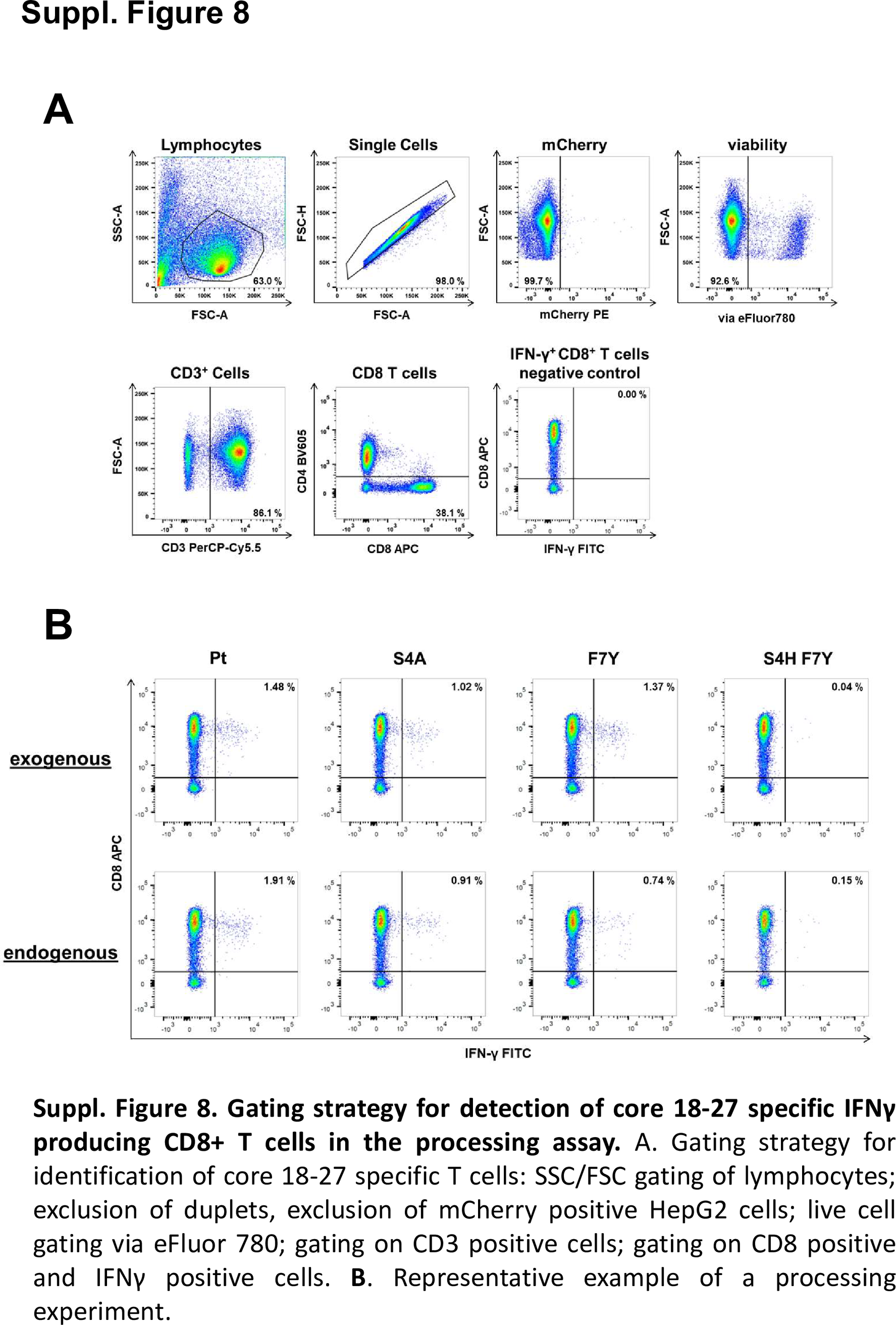

Supplement: Supplementary file 9 [file Image_8.tif]
